# A novel *Arabidopsis* phyllosphere resident *Protomyces* species and a re-examination of genus *Protomyces* based on genome sequence data

**DOI:** 10.1186/s43008-021-00054-2

**Published:** 2021-03-19

**Authors:** Kai Wang, Timo Sipilä, Kirk Overmyer

**Affiliations:** 1 Organismal and Evolutionary Biology Research Program, Faculty of Biological and Environmental Sciences, and Viikki Plant Science Centre, P.O. Box 65, Viikinkaari 1, FI-00014 Helsinki, Finland; 2 Present address: Finnish Institute of Molecular Medicine, University of Helsinki, P.O. Box 20, FI-00014 Helsinki, Finland

**Keywords:** *Taphrinomycotina*, *Taphrinales*, *Protomycetaceae*, Phyllosphere, Phylogenomics, *Brassicaceae*, Average nucleotide identity (ANI), Species delimitation, Carbon source utilization, One new taxon

## Abstract

*Protomyces* is an understudied genus of yeast-like fungi currently defined as phytopathogens of only *Umbelliferae* and *Compositae*. Species relationships and boundaries remain controversial and molecular data are lacking. Of the 82 named *Protomyces,* we found few recent studies and six available cultures. We previously isolated *Protomyces* strains from wild *Arabidopsis thaliana,* a member of *Brassicaceae*, a family distant from accepted *Protomyces* hosts. We previously sequenced the genomes of all available *Protomyces* species, and *P. arabidopsidicola* sp. nov. strain C29, from *Arabidopsis*. Phylogenomics suggests this new species occupied a unique position in the genus. Genomic, morphological, and physiological characteristics distinguished *P. arabidopsidicola* sp. nov. from other *Protomyces*. Nuclear gene phylogenetic marker analysis suggests *actin1* gene DNA sequences could be used with nuclear ribosomal DNA internal transcribed spacer sequences for rapid identification of *Protomyces* species. Previous studies demonstrated *P. arabidopsidicola* sp. nov. could persist on the *Arabidopsis* phyllosphere and *Protomyces* sequences were discovered on *Arabidopsis* at multiple sites in different countries. We conclude that the strain C29 represents a novel *Protomyces* species and propose the name of *P. arabidopsidicola* sp*.* nov. Consequently, we propose that *Protomyces* is not strictly associated only with the previously recognized host plants.

**Supplementary Information:**

The online version contains supplementary material available at 10.1186/s43008-021-00054-2.

## INTRODUCTION

*Protomyces* is a genus of plant pathogenic fungi that cause tumour or gall symptoms within flowers, stems, leaves (especially leaf veins), and petioles on host plants exclusively in the families *Umbelliferae* (*Apiaceae*) and *Compositae* (*Asteraceae*) (Reddy and Kramer 1975; Kurtzman 2011). Members of the genus *Protomyces* have been previously defined based on the morphology of ascospores and vesicles, the host on which they cause disease, and the tissue within the host where they form reproductive structures (Kurtzman 2011; Reddy and Kramer 1975). *Protomyces* species have been stated to cause diseases on “*Apiaceae*, *Compositae*, *Umbelliferae*, and certain other plants” (Kurtzman and Sugiyama 2015; Kurtzman 2011), a claim supported by citations in Tubaki (1957) and Reddy and Kramer (1975). However, closer examination shows that the genus, as currently defined, only contains species pathogenic to plants in *Compositae* and *Umbelliferae* (Table 1).

**Table 1:** Accepted species of *Protomyces.*

Species	Synonym	Host genera	Host family ^a^	Source ^b^	ITS ^c^	Strains ^d^	Genome ^e^
*P. gravidus*	-	*Ambrosia, Bidens*	*Compositae*	A	This study (MK937055)	Y-17093; ATCC 64066	Wang et al. 2019 (QXDP00000000)
*P. inouyei*	-	*Crepis*	*Compositae*	A	This study (MK937056)	CBS 222.57; YB-4354; YB-4365; NBRC 6898; IAM 14512; ATCC 16175; UAMH 1743	Wang et al. 2019 (QXDQ00000000)
*P. lactucaedebilis*	-	*Lactuca debilis*	*Compositae*	A	This study (MK937058)	CBS 223.57; YB-4353; Phaff 12-1054; NBRC 6899	Wang et al. 2019, Riley et al. 2016 (QXDS00000000)
*P. macrosporus*†	*Physoderma gibbosum,* *P. cari*	*Aegopodium, Ammi, Angelica, Anthriscus, Archangelica, Athamanta, Canopodium, Carum, Caucalis, Cherophyllum, Coriandrum, Ferula, Heracleum, Hydrocotyle, Laserpitim, Ligusticum, Meum, Onanthe, Pancicia, Parum, Peucedanum, Pimpinella, Seseli, Ssilaus, Thapsia, Trinia*	*Umbelliferae*	A	This study (MK937059)	Y-12879; PYCC 4286; ATCC 56196	Wang et al. 2019 (QXDT00000000)
*P. pachydermus*	*P. kreuthensis,* *P. centarea,* *P. crepidis,* *P. crepidicola,* *P. crepidis-paludosae,* *P. picridis,* *P. kriegarianus, P. crisii-oleracei*	*Aposeris, Centaurea, Crespis, Criseum, Hyoseris, Hypochaeris, Leontodon, Picris, Taraxacum.*	*Compositae*	A	This study (MK937060)	CBS 224.57; YB-4355; Y-6348; Y-27322; Y-27323, DSM5500; NBRC 6900; IAM 14514; ATCC 90575; MUCL 38937	Wang et al. 2019 (QXDU00000000)
*P. inundatus*	*P. helosciadii, Taphridium innundatus*	*Apium, Daucus, Sium*	*Umbelliferae*	B, C	This study (MK937057)	Y-6349; Y-6802; IAM 6847; ATCC 28148, ATCC 22667; ATCC 28130; ATCC 22666,	Wang et al. 2019 (QXDR00000000)
*P. arabidopsidicola*	-	*Arabidopsis thaliana*	*Brassicaceae*	D	This study (LT602858)	HAMBI3697, DSM 110145	Wang et al. 2019 (QXMI00000000)
*P. burenianus*		*Galinsoga parviflora*	*Compositae*	E	None	None	None
*P. cirsii-oleracei*		*Cirsium oleraceum*	*Compositae*	F	None	None	None
*P. andinus*	*P. giganteus*	*Bidens, Hypochoeris*	*Compositae*	A	None	None	None
*P. burenianus*	-	*Galinsoga*	*Compositae*	A	None	None	None
*P. grandisporus*	-	*Ambrosia*	*Compositae*	A	None	None	None
*P. matricariae*	-	*Matricaria*	*Compositae*	A	None	None	None
*P. sonchi*	-	*Sonchus*	*Compositae*	A	None	None	None

^a^Host plant family: A single family name listed below, both valid names for the same family are listed here; *Compositae* (*Asteraceae*), *Umbelliferae* (*Apiaceae*), or *Brassicaceae* (*Cruciferae*). ^b^ Sources: A, Reddy and Kramer (1975); B, Kurtzman et al. (2011); C, Kurtzman and Robnett (1998); D, Wang et al. (2019); E, Bacigálová (2008); F, Bacigálová et al. (2005). ^c^ Availability of ITS: Internal Transcribed Spacer sequences (with Genebank accession numbers in parentheses) ^d^ Thirty major yeast culture collections were queried for availability of *Protomyces* species in July 2019. The accession numbers of available strains are listed below. Accession numbers that are underlined indicate type strain cultures, which are also the strains used in this study. The full list of queried collections is available in Supplemental file 1. Collections with strains of *Protomyces* species available are listed below: Microbial Domain Biological Resource Centre, Helsinki, Finland (HAMBI; https://www.helsinki.fi/en/infrastructures/biodiversity-collections/infrastructures/microbial-domain-biological-resource-centre-hambi); The German Collection of Microorganisms and Cell Cultures GmbH, Braunschweig, Germany (DSMZ; www.dsmz.de); The Japan Collection of Microorganisms, Koyadai, Tsukuba, Ibaraki, Japan (JCM/IAM; jcm.brc.riken.jp/en/); Biological Resource Center NITE, Chiba, Japan (NBRC; www.nite.go.jp/en/nbrc); CBS-KNAW Fungal Biodiversity Centre, Utrecht, The Netherlands (CBS; http://www.westerdijkinstitute.nl/Collections/); The Portuguese Yeast Culture Collection, Caparica, Portugal (PYCC; http://pycc.bio-aware.com/); Agricultural Research Service Culture Collection, Peoria, IL, USA (Y-/YB-/NRRL; nrrl.ncaur.usda.gov); American Type Culture Collection, Manassas, VA, USA (ATCC; www.atcc.org); University of California Phaff Culture Collection, Davis, CA, USA (UCD-FST; http://phaffcollection.ucdavis.edu); Belgian Coordinated Collection of Microorganisms, Louvain-la-Neuve, Belgium (BCCM/MCLU; http://bccm.belspo.be/); The UAMH Centre for Global Microfungal Biodiversity, Toronto, Canada (UAMH; www.uamh.ca). ^e^ Availability of genome sequencing studies: Wang et al., (2020); Riley et al. (2016), (with Genebank accession numbers in parentheses.) † Type species, *Protomyces* Unger 1833

Some confusion may arise from the dual naming of the plant families *Apiaceae* and *Asteraceae*, which are authorized alternative names for *Umbelliferae* and *Compositae*, respectively (Turland et al. 2018). Tubaki (1957) studied the three *Protomyces* species, *P. inouyei*, *P. lactucaedebilis*, and *P. pachydermus*, which are pathogenic on *Compositae*. Reddy and Kramer’s (1975) taxonomic revision of *Protomycetaceae* indicates that, for all the genera in *Protomycetaceae*, including all *Protomyces* species, the hosts are restricted to *Umbelliferae* and *Compositae*. They accepted 10 species in *Protomyces*, but rejected 61 previously proposed *Protomyces* species, based on their morphology, lack of materials for examination, or association with host plants outside of the families *Umbelliferae* and *Compositae*. Later works expanded the number of known species in the genus *Protomyces* (Bacigálová 2008; Bacigálová et al. 2005; Kurtzman and Robnett 1998; Kurtzman 2011). Bacigálová et al. (2005) list host plants in *Cichoriaceae*, now treated as a subfamily *Cichorioideae* within *Compositae* (Funk and Chan 2009).

There is a lack of molecular data on species of *Protomyces* and other *Protomycetales* genera. Indeed, as previously noted (Kurtzman 2011), there is need for increased efforts in the collection and molecular analysis of these fungi. Our survey of 30 major yeast culture collections (Supplemental file 1; Boundy-Mills et al. 2016) revealed that as of July 2019 there were only six *Protomyces* species with strains available for analysis, including one verified ex-type strain for each of these species (Table 1).

The genus *Protomyces* was established by Unger (1833) with the type species *P. macrosporus*. The sexual cycle is initiated from the diploid hyphal morph (Sugiyama et al. 2006), which occurs primarily during infection in plant tissues. The haploid (asexual) morph is yeast-like, unicellular, reproduces by budding, and is easy to culture. Carotenoid pigments are formed when culturing on artificial growth media (Van Eijk and Roeymans 1982) and colonies are usually yellow to pink. The phylogenetic placement of *Protomyces* has been debated since their first discovery (Kurtzman 2011). *Protomyces* is the type genus of the family *Protomycetaceae*, which also contains genera of currently doubtful phylogenetic relationships and generic boundaries (Reddy and Kramer 1975; Kurtzman 2011), including the soil and insect associated *Saitoella,* where the genome of one species has been sequenced (Nishida et al. 2011)*,* and the *Protomyces*-like plant pathogens, *Burenia, Protomycopsis, Taphridium,* and *Volkartia*, for which there are currently no cultures or molecular data available*.*

Carbon source utilization profiles remain a quick and useful additional tool for the identification of yeast species (Kurtzman et al. 2011) and recent fungal genome sequencing projects have begun to address the relationships between biochemical traits and gene content (Riley et al. 2016). The use of molecular analysis unambiguously placed *Protomyces* in the ascomycete subphylum *Taphrinomycotina*, as a sister clade to the genus *Taphrina* in the order *Taphrinales* (Walker 1985; Nishida and Sugiyama 1994; Kurtzman and Robnett 1998; James et al. 2006; Sugiyama et al. 2006; Hibbett et al. 2007; Kurtzman 1993a). It has been noted in the closely related genus *Taphrina* that the nuclear ribosomal DNA (nrDNA) 26S large-subunit D1/D2 domain (D1/D2) and especially nrDNA internal transcribed spacer (ITS) markers gave resolution to the genus level, but some species could not be resolved (Rodrigues and Fonseca 2003). In general, there is a need in some fungal taxa to develop lineage specific secondary phylogenetic markers to achieve reliable species level identification; DNA markers, such as actin, translation elongation factors, ribosomal polymerase subunits and tubulin, have been described and applied for this purpose (Stielow et al. 2015).

It has been suggested that members of *Protomyces,* its sister genus *Taphrina,* and some other members of *Taphrinales*, may have retained the life-styles of early *Ascomycota*, due to their many ancestral features and basidiomycete-like traits, such as high genomic GC content (Sugiyama et al. 1996; Wang et al. 2019), thick walled “chlamydospore” reproductive or resting cells (de Bary and Garnsey 1887; Mix 1924), basidiospore-like naked asci (Sadebeck 1884; Lohwag 1934), enteroblastic budding pattern (Sugiyama et al. 1996; Von Arx and Weijman 1979), Q-10 ubiquinone system (Sugiyama et al. 2006), and the presence of a putative dual hybrid histidine kinase (Wang et al. 2019). These similarities are also illustrated by the many instances, in which species within the *Taphrinales* have been misclassified amongst the basidiomycetes, or vice versa (Piepenbring and Bauer 1997; de Bary and Garnsey 1887; Reddy and Kramer 1975; Nishida and Sugiyama 1995). Due to their phylogenetic position and these characteristics, these species are of considerable evolutionary interest.

Here we describe a *Protomyces* strain C29, isolated from the phyllosphere of wild *Arabidopsis thaliana*, as a new *Protomyces* species, *P. arabidopsidicola*. The delimitation of the genus *Protomyces* and boundaries of species within it are also examined here in the light of new genome sequencing data.

## MATERIALS AND METHODS

For comparison, confirmed ex-type strains of six *Protomyces* species (Table 1) were obtained from the USDA ARS culture collection (NRRL; https://nrrl.ncaur.usda.gov/). Species and strains used are: *Protomyces arabidopsidicola* sp. nov. strains C29, C2–11, and C2–15; *P. gravidus* strain Y-17093; *P. inouyei* strain YB-4354; *P. inundatus* strain Y-6349; *P. lactucaedebilis* strain YB-4353; *P. macrosporus* strain Y-1287; *P. pachydermus* strain YB-4355*.* The isolation of the culture *P. arabidopsidicola* strains C29, C2–11, and C2–15, from the leaf surface of healthy wild-growing *Arabidopsis thaliana*, in Helsinki, Finland, was described by Wang et al. (2016). Culture conditions, DNA extraction, PCR amplification, and DNA sequencing, were as described elsewhere (Wang et al. 2019; Wang et al. 2016). Average nucleotide identity (ANI) and average amino-acid identity (AAI) values of *Protomyces* genomes or proteomes were calculated using the online tool ANI/AAI-Matrix (Rodriguez-R and Konstantinidis 2016). Percent nucleotide identity of nrDNA and protein coding phylogenetic marker genes was determined using pairwise BLASTn (megablast) alignments using the multiple alignment tool at NCBI (National Center for Biotechnology Information; www.ncbi.nlm.nih.gov). Marker gene sequences for all species are available using the accession numbers listed below. Growth assays at low temperature were previously reported in Wang et al. (2019). Yeast cell size and morphological characterization was conducted by photographing three-day-old or two-week-old cultures with a LEICA 2500 microscope (www.leica-microsystems.com) or two-week-old colonies with a LEICA MZ10F stereo microscope. Cells and colonies were cultivated on YPG (yeast-extract, peptone, glucose). For cell size measurements, yeast cells were mounted on a slide in water for examination. Cell length and width were measured from photomicrographs with imageJ software (https://imagej.nih.gov/ij/). Carbon assimilation patterns among seven species were tested with API 50 CH strips (bioMerieux SA; www.biomerieux.com) cultured at 21 °C for 7 d, according to the manufacturer’s instructions.

The genome sequences and annotations of *Saitoella complicata* Saico1, *Saccharomyces cerevisiae* GCF000146045.2 R64, *Schizosaccharomyces pombe* GCF000002945.1, *Pneumocystis murina* B123, *Taphrina populina* JCM22190, and *Neolecta irregularis* DAH-3 were downloaded from NCBI (www.ncbi.nlm.nih.gov/). The phylogenetic tree with species representing *Taphrinomycotina* classes *Neolectomycetes*, *Pneumocystidomycetes*, *Schizosaccharomycetes*, and *Taphrinomycetes* was built with 636 single-copy protein sequences from genome annotations. *Saccharomyces cerevisiae,* representing *Saccharomycotina,* was utilized as an outgroup. Conserved single-copy protein sequences were identified with orthofinder (Emms and Kelly 2015). Alignment quality was controlled by applying sequence scores > = 0.8 in MAFFT analysis with Guidance2 (Sela et al. 2015). Multiple sequences were concatenated with FASconCAT_V1.0 (Kück and Meusemann 2010). The randomized axelerated maximum likelihood method RAxML (Stamatakis 2014) and rapid bootstrapping (100x) (Stamatakis 2014) were employed for building the genome-wide tree, and (1000x) for nuclear DNA marker trees. Phylogenetic trees using the nuclear DNA markers; actin1 (*act1),* second largest subunit of RNA polymerase II (*rbp2)*, large subunit of RNA polymerase II (*rbp1)*, transcription elongation factor 1 (*tef1)* and tubulin beta chain (*tub2)* were aligned by ClustalX2.1 (Larkin et al. 2007). Sequences of DNA marker gene orthologs in *Protomyces* and *Taphrina* were discovered using local BLAST searches of their genomes using *Schizosaccharomyces* sequences as query. All phylogenetic trees were viewed and edited with iTOL (Letunic and Bork 2016). Trees based on the Bayesian inference method were constructed utilizing the MrBayes software package (Ronquist et al. 2012), selecting the general time reversible model and invgamma. Two independent analyses were started simultaneously with 10,000 generations and four chains set for the analysis run.

The sequences of proteins known to be involved in carbohydrate utilization in model yeast species were collected from NCBI and UniProt (https://www.uniprot.org/). Characterized protein sequences of key enzymes were selected as query sequences. Sequences used as search queries are listed in Supplementary File 2. A local installation of BLASTp was applied for searching enzyme hits in *Protomyces* genomes. Selection criteria are E value <1e^− 30^ and protein hits were manually curated to avoid duplicates. All analyses above were performed using CSC (https://www.csc.fi/) computing resources in Linux.

## RESULTS

### Evidence of the new *Protomyces* species

We previously isolated a novel *Protomyces* strain, strain C29, from the phylloplane of healthy individuals of thale cress (*Arabidopsis thaliana*) (Wang et al. 2016) growing in the wild in Helsinki, Finland. The strain C29, a member of operational taxonomic unit1 (OTU1), was identified as belonging to the genus *Protomyces* by BLAST searches with ITS sequences (Genbank acc. LT602858) showing the highest similarity (96%) to *P. inouyei* (Wang et al. 2016). Two other identical strains (C2–11 and SC2–15) belonging to OTU1 based on identical ITS sequences where also isolated. One additional strain (strain A14) belonging to the genus *Protomyces* in OTU2, with an ITS sequence (Genbank acc. LT602859) similar but not identical to OTU1, was isolated from a distinct sample, giving four strains from two independent *Arabidopsis* individuals from two distinct locations within the common sampling area (Wang et al. 2016). The genome of the new species, named as *P. arabidopsidicola* below, and the genomes of six other *Protomyces* species were previously sequenced and subjected to comparative analysis (Wang et al. 2019). These six sequenced species represent all of the *Protomyces* species for which strains are currently available in culture collections (Table 1). All sequenced *Protomyces* species have small genome sizes (11.5–14.1 Mbp) with 50.9–52.8% GC content. Our previous work (Wang et al. 2019) focused on the ecology of *P. arabidopsidicola*, its interactions with *Arabidopsis,* and comparison of the features of the seven sequenced genomes. Here we focus on the description and naming of the novel species as well as the phylogenetic implications of our *Protomyces* genome sequencing results (Wang et al. 2019).

**Fig. 1:**
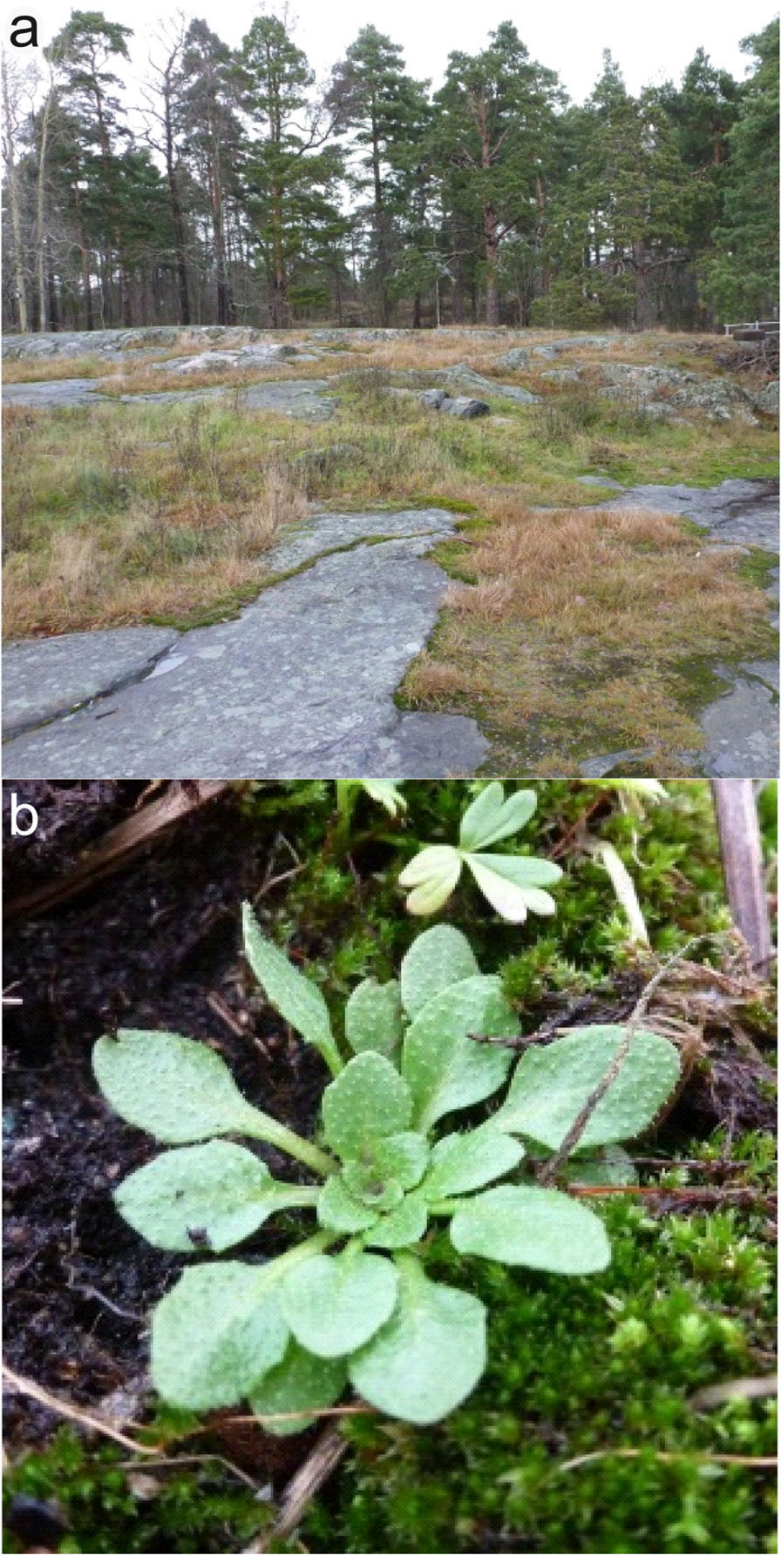
Isolation environment and host. **a** The environment from which *Protomyces arabidopsidicola* sp. nov. was isolated. **b** A typical example of healthy wild thale cress (*Arabidopsis thaliana)* plants that were collected for yeast isolation

**Fig. 2:**
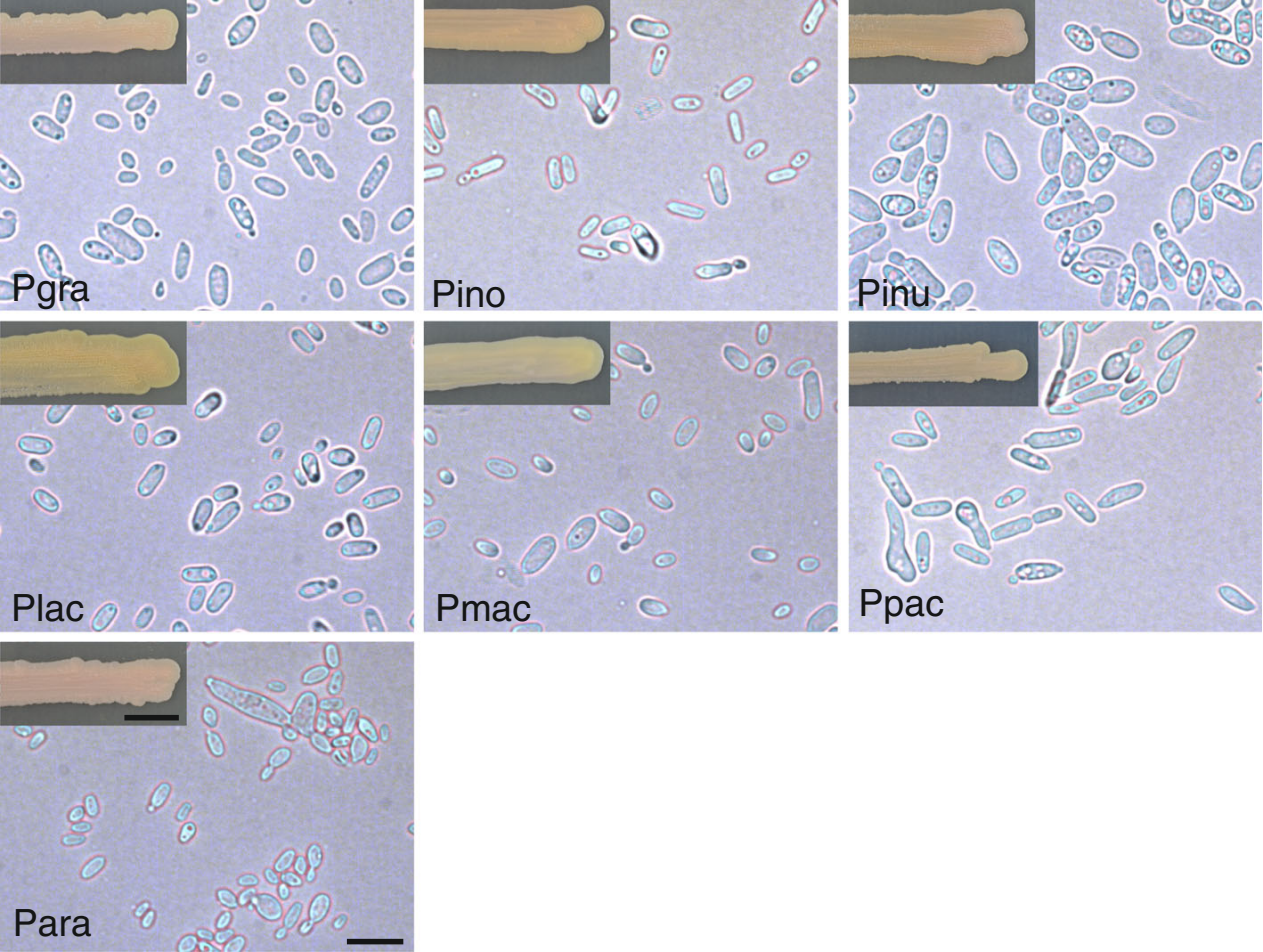
Morphology of *Protomyces species*. Morphology of yeast cells and colonies of *Protomyces* species including *Protomyces arabidopsidicola.* Yeasts were cultured in YPG (peptone, yeast-extract, glucose) agar (2%) medium. Photos of colonies taken 2 weeks after inoculation. Photos of cells were taken 3 days after inoculation. Pgra, *P. gravidus*; Pino, *P. inouyei*; Pinu, *P. inundatus*; Plac, *P. lactucaedebilis;* Pmac, *P. macrosporus*; Ppac, *P. pachydermus*; Para: *P*. *arabidopsidicola*. Scale bars, 10 μm for yeast cells and 1 cm for colonies, are valid for all panels

In Fig. 1, the typical peat moss and stone environmental conditions of sites where *Arabidopsis* samples were collected for isolation is shown (Fig. 1a) as well as a representative individual of the healthy *Arabidopsis* plants collected in their native habitat (Fig. 1b). Yeast cell sizes (Table 2) as well as cell and colony morphology (Fig. 2) of all seven *Protomyces* species were analysed, where the cell size of *P. arabidopsidicola* was significantly shorter than its closest relative *P. inouyei*, suggesting it is a distinct species.

**Table 2:** Yeast cell sizes

Age	Species	Mean size ± SD (μm)	Size range (μm)	Reference*
3 d	*P. arabidopsidicola*	4.8 ± 1.6 × 2.7 ± 0.6	2.0–12.4 × 1.4–5.6	NA
	*P. gravidus*	5.2 ± 1.3 × 3.0 ± 0.6	2.7–9.6 × 1.4–5.9	2.5–10 × 2.5–4
	*P. inouyei*	6.3 ± 1.6 × 2.6 ± 0.4	3.2–11.4 × 1.5–4.2	2.5–10 × 2–4
	*P. inundates*	7.5 ± 2.1 × 3.8 ± 0.6	3.8–14.2 × 1.1–5.4	3.7–9 × 2–4.7
	*P. lactucaedebilis*	5.4 ± 1.5 × 2.7 ± 0.4	3.0–10.8 × 1.6–4.0	3.5–9 × 2.5–5
	*P. macrosporus*	5.2 ± 1.6 × 2.9 ± 0.5	2.3–13.0 × 1.7–4.2	3–7 × 2.5–4
	*P. pachydermus*	7.4 ± 2.1 × 2.9 ± 0.6	3.6–13.6 × 1.6–5.5	3–8 × 2.5–4
14 d	*P. arabidopsidicola*	4.7 ± 1.8 × 2.6 ± 0.8	2.4–14.1 × 1.6–5.7	NA
	*P. gravidus*	5.4 ± 1.4 × 2.6 ± 0.5	3.2–8.2 × 1.4–3.8	
	*P. inouyei*	4.7 ± 1.3 × 2.7 ± 1.1	2.6–10.8 × 1.1–6.5	
	*P. inundates*	8.7 ± 3.8 × 5.6 ± 2.7	3.6–18.7 × 1.9–16.2	
	*P. lactucaedebilis*	5.2 ± 1.5 × 2.9 ± 0.8	2.8–9.7 × 1.5–5.0	
	*P. macrosporus*	4.9 ± 1.3 × 2.7 ± 1.0	3.0–8.5 × 1.6–8.1	
	*P. pachydermus*	6.4 ± 2.6 × 3.0 ± 0.5	3.8–20.8 × 1.9–4.0	

Measurements were from 3 d and 14 d cells of *Protomyces* species. Yeast cells were cultured in YPG agar plates. Cell images were captured by LEICA 2500 microscope with a LEICA DFC490 camera. Cell length and width were measured with imageJ software. Two independent cultures were applied in the statistics, with around 100 cells sampled in each biological repeat. The yeast cells of *P. arabidopsidicola* had a significantly shorter length than its closest relative *P. inouyei* (*p* < 0.05) by one way ANOVA + Tukey HSD. NA: not available. “Reference*”: is the published size range for each species according to Kurtzman (2011).

**Fig. 3:**
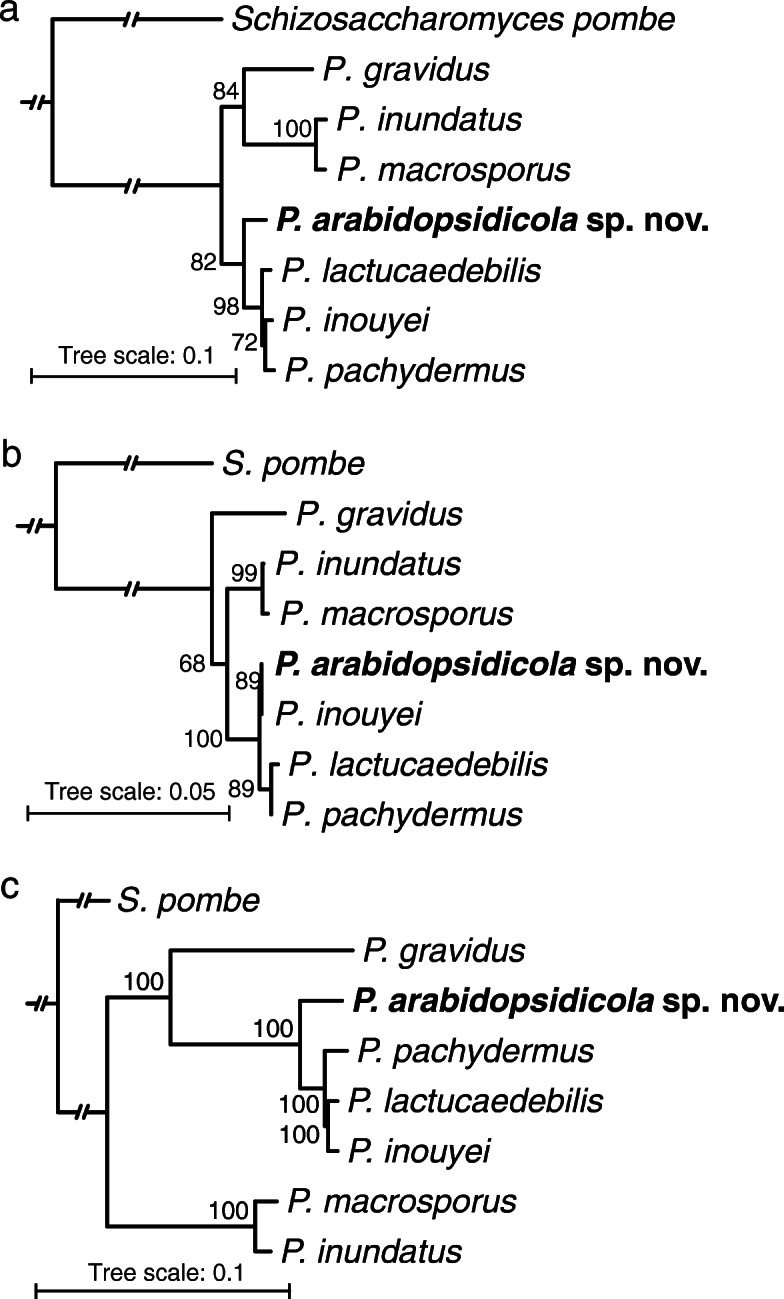
Phylogenetic analysis of the genus *Protomyces*. Phylogenetic tree built by ITS (**a**), D1D2 (**b**) and genome-wide sequences (**c**), with maximum likelihood method. **a** ITS and **b** D1D2 sequences were aligned with ClustalX2. Bootstraping (1000x) was conducted and best-scoring ML tree were produced with randomized axelerated maximum likelihood (RAxML) rapid bootstrapping program. Bootstrap support values (%) are indicated at each node. **c** RAxML with rapid bootstrapping (100x) were chosen for constructing the maximum likelihood tree utilizing 1670 concatenated single-copy conserved protein sequences from whole genome sequence data. Alignment quality control of single-copy conserved proteins was achieved by applying sequence scores > = 0.8 in MAFFT analysis using Guidance2. Multiple aligned sequences of each species/strain were concatenated using FASconCAT_V1.0 and bootstrap values (%) are indicated at the nodes. The output .tre files were viewed with online tool iTOL. In all phylogenies *Schizosaccharomyces pombe* was used as an outgroup. For the same phylogenies built using the Bayesian inference method, see Supplemental Fig. 1

In order to reveal the evolutionary relationships and species delimitation within the genus, we built phylogenetic trees using various markers; specifically, DNA sequences of the nrDNA D1/D2 domain, the full ITS DNA sequences (ITS1-5S rDNA-ITS2), and conserved protein coding sequences from the genome sequencing data of *P. arabidopsidicola* and the other *Protomyces* species. All phylogenetic trees were constructed with the Maximum Likelihood (Fig. 3) and the Bayesian inference methods (Supplemental Fig. 1), which both yielded similar results and support the same conclusions. The ITS marker differentiated all strains at the species level, but with low support for some nodes (Fig. 3 a). D1/D2 trees offered poor species resolution and were poorly supported (Fig. 3 b). In order to quantitatively support the differences seen in these phylogenetic trees, we calculated the percent nucleotide identity between all the examined *Protomyces* species for the ITS and D1/D2 sequences (Table 3 a, b)

**Fig. 4:**
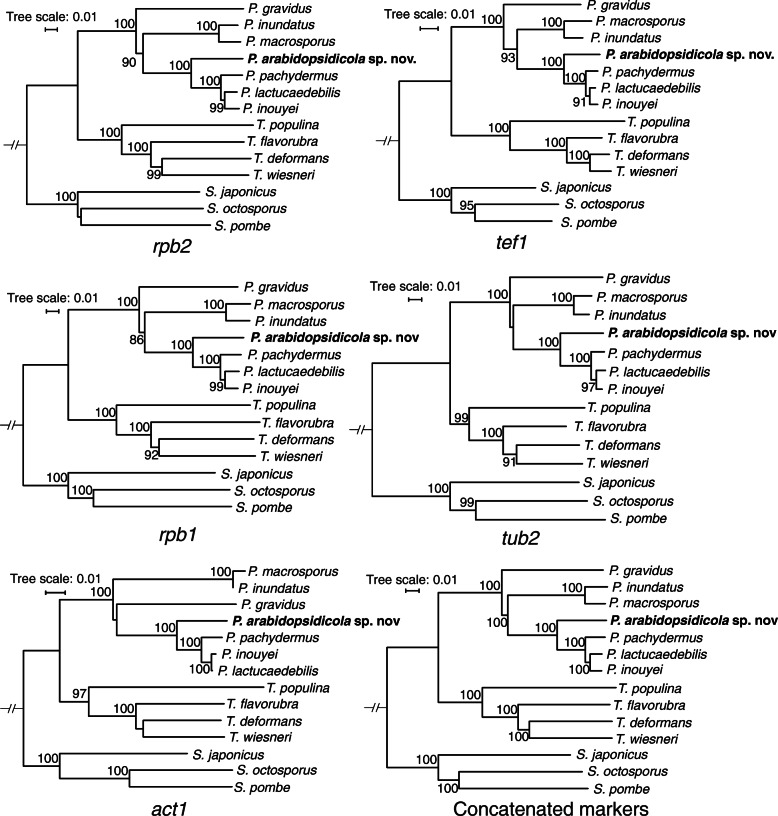
Nuclear marker gene phylogenetic trees. Phylogenetic trees of species in the genera *Protomyces* and *Taphrina* with the nuclear gene markers *rbp2* (RNA polymerase subunit 2), *tef1* (translation elongation factor 1), *rbp1* (RNA polymerase subunit 1), *tub2* (tubulin beta), *act1* (actin 1) and concatenated sequence of the five nuclear marker genes above. Protein sequences from the respective marker genes in *Schizosaccharomyces pombe* were used as a query for BLASTp searches against protein sequences from *Protomyces* and *Taphrina* genome annotations. DNA sequences of each marker were then collected and aligned with ClustalX2 to construct neighbour-joining trees with 1000 bootstraps. Bootstrap support values (%) are indicated at each node. *Schizosaccharomyces pombe* was used as an outgroup

These results taken together suggest that the true diversity of the genus *Protomyces* is not captured or supported in the phylogenies utilizing the commonly used D1/D2 or ITS markers. Therefore, other methods are required to resolve the phylogeny of species in the genus. To this end, we utilized genome sequencing data and performed genome-wide phylogenetic analysis with the Maximum-Likelihood method using RAxML software (Stamatakis 2014) and the Bayesian inference method utilizing 1670 single-copy protein sequences that were common to these seven genomes. Clearly, *P. arabidopsidicola* occupies a unique position in a monophyletic clade with the six other *Protomyces* species, indicating it is a novel species of *Protomyces* most closely related to a clade composed of *P. inouyei*, *P. lactucaedebilis*, and *P. pachydermus* (Fig. 3, c). The percent identity of ITS marker genes also support that this is a new species (Table 3). As genomic sequencing is not practical for rapid species identification, we tested five nuclear genes *rbp2*, *tef1*, *rbp1, tub2,* and *act1* as potential secondary lineage-specific phylogenetic DNA markers, both individually and together as a single concatenated sequence (Fig. 4). All of these nuclear DNA markers resolved *P. arabidopsidicola* as distinct from other *Protomyces* species analyzed, providing additional evidence at the DNA level that *P. arabidopsidicola* represents a novel species of *Protomyces* (Fig. 4). All markers performed reasonably well at resolving species in the genus (Fig. 4). However, only the *act1* tree topology closely resembled whole genome data, thus percent nucleotide identity for the *act1* gene was calculated (Table 3 c).

**Table 3:** Percent nucleotide identity for three phylogenetic marker genes

	Pinu	Pmac	Pgra	Plac	Pino	Ppac	Para
(a) ITS							
Pinu	100						
Pmac	98.1	100					
Pgra	90.3	89.8	100				
Plac	90.6	90.8	90.1	100			
Pino	90.9	91.0	91.1	98.3	100		
Ppac	90.4	91.4	90.2	98.1	98.2	100	
Para	91.7	91.7	90.8	95.7	96.2	95.5	100
(b) D1/D2							
Pinu	100						
Pmac	99.8	100					
Pgra	96.9	96.7	100				
Plac	97.6	97.4	96.2	100			
Pino	97.9	97.7	96.2	99.7	100		
Ppac	97.7	97.6	96.4	99.5	99.5	100	
Para	98.1	97.9	96.7	99.5	99.5	99.3	100
(c) Act1							
Pinu	100						
Pmac	98.8	100					
Pgra	87.4	86.8	100				
Plac	88.2	88.2	88.8	100			
Pino	88.3	88.3	88.8	99.7	100		
Ppac	87.9	87.9	88.9	98.4	98.2	100	
Para	87.6	87.8	88.0	95.7	96.7	95.1	100

Percent nucleotide identity between *Protomyces* species determined by pairwise BLASTn (megablast) alignments for the ribosomal genes ITS (a) and D1/D2 (b), and the protein coding gene, Actin1 (Act1) (c). Species name abbreviations: *Protomyces inundatus* (Pinu), *P. macrosporus* (Pmac), *P. gravidus* (Pgra), *P. lactucaedebilis* (Plac), *P. inouyei* (Pino), *P. pachydermus* (Ppac), and *P. arabidopsidicola* (Para).

In addition to phylogenetic analysis, comparisons of whole genome ANI and AAI between *P. arabidopsidicola* and the other six species were used as evidence to define species borders. ANI and AAI values were between 77.5 to 85.6% and 71.9 to 90.6%, respectively (Table 4). The low levels of ANI (≤95%) and AAI provide further evidence that *P. arabidopsidicola* is a new species distinct from the other six *Protomyces* species included.

**Table 4:** ANI and AAI values between *Protomyces* species genomes

	Pinu	Pmac	Pgra	Plac	Pino	Ppac	Para
Pinu	–	94.7	71.4	71.9	71.8	71.9	72.0
Pmac	**92.0**	–	71.3	71.8	71.7	71.9	71.9
Pgra	**77.9**	**77.8**	–	74.2	74.1	74.1	74.2
Plac	**78.0**	**78.3**	**77.6**	–	97.3	95.6	90.6
Pino	**78.0**	**77.9**	**77.6**	**96.0**	–	95.6	90.6
Ppac	**78.0**	**77.8**	**77.5**	**93.6**	**93.6**	–	90.1
Para	**77.9**	**78.1**	**77.5**	**85.6**	**85.5**	**85.0**	–

ANI (average nucleotide identity) and AAI (average amino-acid identity) values among *Protomyces* species. Genome and annotation matrix were applied with online tool ANI/AAI-Matrix using data from (Wang et al. 2019). The lower matrix shows the ANI values (in bold) and the upper matrix indicates the AAI values (in plain text). Species name abbreviations: *Protomyces inundatus* (Pinu), *P. macrosporus* (Pmac), *P. gravidus* (Pgra), *P. lactucaedebilis* (Plac), *P. inouyei* (Pino), *P. pachydermus* (Ppac), and *P. arabidopsidicola* (Para).

**Fig. 5:**
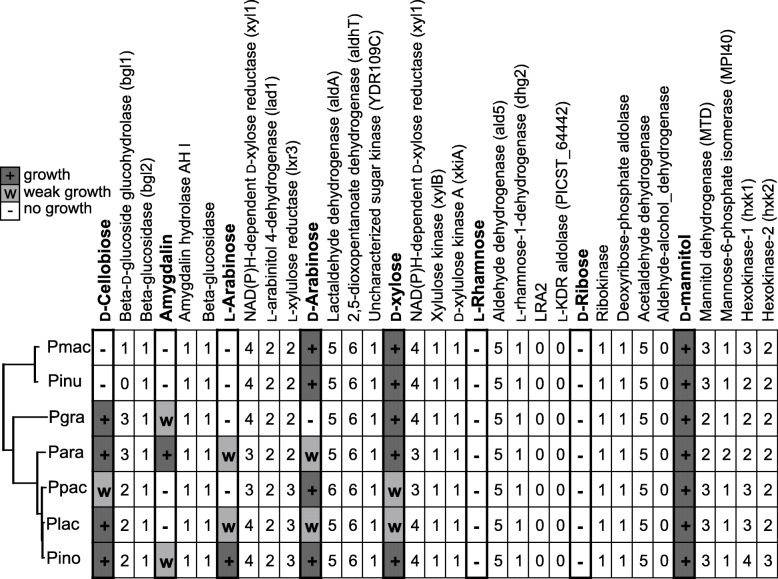
Carbon assimilation traits versus nuclear genes. Distribution of carbon assimilation patterns and related enzymes in the genus *Protomyces*. Yeast cells were grown in api® 50 CH strips with a starting OD = 0.1, and were visually scored for growth on day seven (Table 5). Growth patterns were classified as growth (+), weak growth (w) and no growth (−). Numbers indicate the enzyme hits found for each carbon assimilation pathway. Genes encoding *Protomyces* carbon metabolism enzymes were identified using sequences of conserved homologs that have been characterized in model yeast species, see Supplemental file 2 for the protein sequences used as BLAST queries. Selection criteria for BLAST results is an E value <1e^−30^. Species name abbreviations used: *Protomyces inundatus* (Pinu), *P. macrosporus* (Pmac), *P. gravidus* (Pgra), *P. lactucaedebilis* (Plac), *P. inouyei* (Pino), *P. pachydermus* (Ppac), and *P. arabidopsidicola* strain C29 (Para).

Carbon assimilation profiles of *P. arabidopsidicola* and the other six *Protomyces* species were determined (Table 5). These data demonstrate that each species utilized a unique pattern of carbon sources. *P. arabidopsidicola* exhibited a distinct profile of carbon utilization traits, especially for D-cellobiose, amygdalin, L-arabinose and D-arabinose (Table 5) further distinguishing it from *P. inouyei*. Based on this evidence, a new diagnostic key of seven species is provided below. Utilizing the genome annotations of seven *Protomyces* species, we compared the presence of genes known to be involved in carbon source metabolism with selected metabolic traits (Fig. 5), including those that distinguish the *Protomyces* species in this study (Table 5). For some traits such as utilization of D-cellobiose, L-rhamnose, D-ribose, and D-mannitol, the presence or absence of full pathways and the number of paralogs correlated with metabolic phenotypes (Fig. 5). However, for amygdalin, L-arabinose, D-arabinose, and D-xylose, the genes encoding these enzymes were generally highly conserved and thus do not correlate well with the inability of some species to utilize these carbon sources (Fig. 5).

**Table 5:** Carbon assimilation patterns of *Protomyces* species

Carbon source	Pgra	Pino	Pinu	Plac	Pmac	Ppac	Para
Glycerol	**+**	**+**	**+**	**+**	**+**	**+**	**+**
Erythritol	**–**	**–**	**–**	**–**	**–**	**–**	**–**
**D-Arabinose**	**–**	**+**	**+**	**w**	**+**	**+**	**W**
**L-Arabinose**	**–**	**+**	**–**	**w**	**–**	**–**	**W**
D-Ribose	**–**	**–**	**–**	**–**	**–**	**–**	**–**
**D-Xylose**	**+**	**+**	**+**	**w**	**+**	**w**	**+**
L- Xylose	**–**	**–**	**–**	**–**	**–**	**–**	**–**
**D-Adonitol**	**–**	**+**	**–**	**–**	**–**	**–**	**–**
Methyl-βD- xylopyranoside	**–**	**–**	**–**	**–**	**–**	**–**	**–**
D-Galactose	**–**	**–**	**–**	**–**	**–**	**–**	**–**
D-Glucose	**+**	**+**	**+**	**+**	**+**	**+**	**+**
D-Fructose	**+**	**+**	**+**	**+**	**+**	**+**	**+**
D-Mannose	**+**	**+**	**+**	**+**	**+**	**+**	**+**
L-Sorbose	**–**	**–**	**–**	**–**	**–**	**–**	**–**
**L-Rhamnose**	**–**	**+**	**–**	**–**	**–**	**–**	**–**
Dulcitol	**–**	**–**	**–**	**–**	**–**	**–**	**–**
**Inositol**	**–**	**+**	**–**	**–**	**–**	**–**	**–**
D-Mannitol	**+**	**+**	**+**	**+**	**+**	**+**	**+**
**D-Sorbitol**	**–**	**w**	**–**	**+**	**w**	**w**	**+**
**Methyl-αD-Mannopyranoside**	**–**	**+**	**–**	**–**	**–**	**–**	**w**
**Methyl-αD-Glucopranoside**	**–**	**w**	**+**	**+**	**–**	**w**	**w**
**N-Acetylglucosamine**	**–**	**+**	**–**	**–**	**–**	**–**	**–**
**Amygdalin**	**w**	**w**	**–**	**–**	**–**	**–**	**+**
**Arbutin**	**w**	**+**	**–**	**–**	**–**	**–**	**+**
**Esculin ferric citrate**	**+**	**+**	**–**	**+**	**–**	**–**	**+**
**Salicin**	**w**	**+**	**–**	**–**	**–**	**–**	**+**
**D-Cellobiose**	**+**	**+**	**–**	**+**	**–**	**w**	**+**
D-Maltose	**+**	**+**	**+**	**+**	**+**	**+**	**+**
D-Lactose	**–**	**–**	**–**	**–**	**–**	**–**	**–**
D-Melibiose	**–**	**–**	**–**	**–**	**–**	**–**	**–**
D-Saccharose (sucrose)	**+**	**+**	**+**	**+**	**+**	**+**	**+**
D-Trehalose	**+**	**+**	**+**	**+**	**+**	**+**	**+**
**Inulin**	**+**	**+**	**+**	**+**	**–**	**w**	**–**
**D-Melezitose**	**+**	**+**	**+**	**+**	**+**	**+**	**w**
D-Raffinose	**+**	**+**	**+**	**+**	**+**	**+**	**+**
Amidon (starch)	**+**	**+**	**+**	**+**	**+**	**+**	**+**
**Glycogen**	**w**	**–**	**+**	**–**	**–**	**w**	**–**
**Xylitol**	**w**	**+**	**+**	**+**	**+**	**+**	**+**
**Gentiobiose**	**+**	**+**	**–**	**+**	**–**	**–**	**+**
D-Turanose	**+**	**+**	**+**	**+**	**+**	**+**	**+**
**D-Lyxose**	**w**	**w**	**+**	**–**	**+**	**w**	**–**
D-Tagatose	**–**	**–**	**–**	**–**	**–**	**–**	**–**
D-Fucose	**–**	**–**	**–**	**–**	**–**	**–**	**–**
L-Fucose	**–**	**–**	**–**	**–**	**–**	**–**	**–**
**D-Arabitol**	**+**	**+**	**–**	**+**	**+**	**+**	**+**
**L-Arabitol**	**w**	**w**	**+**	**–**	**–**	**–**	**–**
Potassium gluconate	**–**	**–**	**–**	**–**	**–**	**–**	**–**
**Potassium 2-Ketogluconate**	**w**	**+**	**–**	**+**	**w**	**–**	**w**
**Potassium 5-Ketogluconate**	**–**	**–**	**–**	**–**	**–**	**w**	**–**

Carbon assimilation was tested using 50 CH strips (biomerieuxdirect.com). Key to growth symbols: +, positive, w, weakly positive, −, negative. Abbreviations: Pgra, *P. gravidus*; Pino, *P. inouyei*; Pinu, *P. inundatus*; Plac, *P. lactucaedebilis*; Pmac, *P. macrosporus*; Ppac, *P. pachydermus*; Para, *P. arabidopsidicola*. Carbon sources listed in bold text are used differentially by these *Protomyces* species

### Phylogenomics

**Fig. 6:**
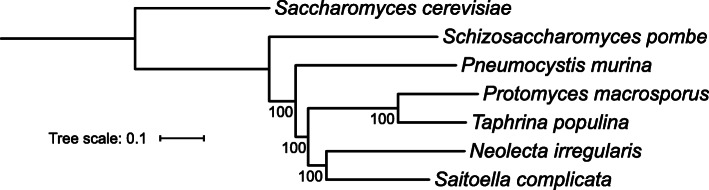
Phylogeny of the subphylum *Taphrinomycotina*. Maximum likelihood phylogenetic tree of representative species in the subphylum *Taphrinomycotina*. Tree was built using 636 single-copy protein sequences that were common to all species used. Alignment quality control of single-copy conserved proteins was achieved by applying sequence scores > = 0.8 in MAFFT analysis using Guidance2. *Saccharomyces cerevisiae* was used as an outgroup. Multiple aligned sequences of each species were concatenated into a single long sequence using FASconCAT_V1.0. The randomized axelerated maximum likelihood method (RAxML) and rapid Bootstrapping (100x) were applied for constructing the maximum-likelihood phylogenetic tree. Bootstrap support values (%) are shown at each node. The output files were viewed with online tool iTOL. For the same phylogeny built using the Bayesian inference method, see Supplemental Fig. 2

The genus *Protomyces* resides within the *Ascomycota* subphylum *Taphrinomycotina*, class *Taphrinomycetes*, order *Taphrinales*, and family *Protomycetaceae*. The placement of the genus *Protomyces* has been problematic since its discovery. Relationships between genera are not well supported within the subphylum *Taphrinomycotina* (Kurtzman et al. 2011). Genome-wide phylogenetic trees constructed using both the Maximum-Likelihood (Fig. 6) and Bayesian inference (Supplemental Fig. 2) methods with concatenated single-copy conserved proteins from species representing each family within *Taphrinomycotina* were well supported*.* These confirmed the placement of *Protomyces* within this subphylum*,* as well as suggesting novel relationships between other genera within the subphylum.

### *Protomyces inouyei* and *P. lactucaedebilis*

**Fig. 7:**
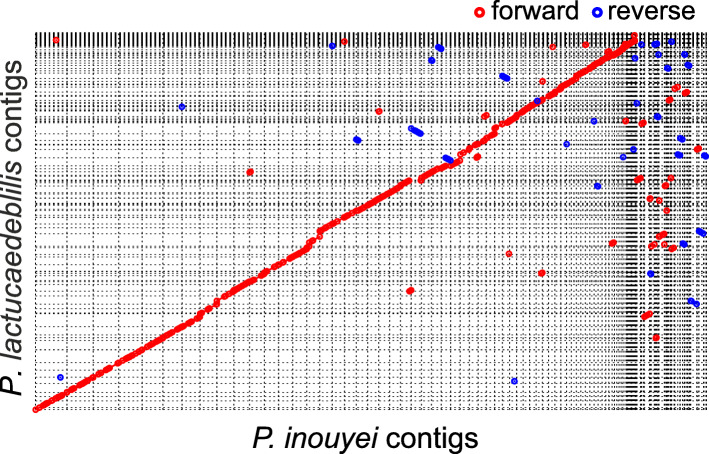
Genome synteny of *Protomyces inouyei* and *P. lactucaedebilis*. Dot-plots of whole genome alignment between *Protomyces inouyei* and *P. lactucaedebilis* contigs. Mummer 4.0.0 beta2 (nucmer) was applied for genome assembly comparison with default settings. Forward alignment is shown in red and reverse alignment in blue. Optimal co-linear order of contigs was shaped with mummerplot (parameter --fat). Mummerplot output ps files were viewed and edited with CorelDraw 2018

Previous treatments of *Protomyces* (Kurtzman 2011), which utilized the same six species we have sequenced and analyzed here, were unable to definitively conclude that these species were all genetically distinct. Our results (Table 4) clearly demonstrate that most are distinct species. However, the two species, *P. inouyei* and *P. lactucaedebilis,* may not be distinct given their > 96% ANI and > 97% AAI values at the whole genome level (Table 4). The evolutionary distance between them is small in the phylogenetic trees built with all makers tested (Figs. 3, 4, Table 3). Additionally, comparisons of genomic assemblies indicated a very high level of synteny between the genomes of *P. inouyei* and *P. lactucaedebilis* (Fig. 7). These data suggest that *P. inouyei* and *P. lactucaedebilis* may be strains of the same species, rather than distinct species.

### Dichotomous key to *Protomyces* species

The diagnostic key is based on carbon assimilation patterns from D-adonitol, Methyl-αD-mannopyranoside, L-arabinose, salicin, D-cellobiose and inulin.

The diagnostic key is based on carbon assimilation patterns from D-adonitol, Methyl-αD-mannopyranoside, L-arabinose, salicin, D-cellobiose and inulin.

**Table:** 

1 D-adonitol is assimilated	***P. inouyei***
D-adonitol is not assimilated	2
2 (1) Ethyl-αD-mannopyranoside is assimilated	***P. arabidopsidicola***
Methyl-αD-mannopyranoside is not assimilated	3
3 (2) L-arabinose is assimilated	***P. lactucaedebilis***
L-arabinose is not assimilated	4
4 (3) Salicin is assimilated	***P. gravidus***
Salicin is not assimilated	5
5 (4) D-cellobiose is assimilated	***P. pachydermus***
D-cellobiose is not assimilated	6
6 (5) Inulin is assimilated	***P. inundatus***
Inulin is not assimilated	***P. macrosporus***

## TAXONOMY

***Protomyces arabidopsidicola*** Kai Wang & Overmyer, **sp**. **nov**.

*MycoBank:* MB 830646.

*Etymology*: The epithet refers to the host plant (*Arabidopsis thaliana*)*,* from which the fungus was isolated (“the *Arabidopsis*-inhabiting *Protomyces*”.)

*Diagnosis:* Molecularly differentiated from all other *Protomyces* species based on ITS and Actin1, but not D1/D2, gene sequences. Physiologically differentiated from *P. inouyei* based on its inability to utilize D-adonitol, D-lyxose, inulin, inositol, L-rhamnose, or L-arabitol. Further differentiated from other *Protomyces* species (*P. gravidus*, *P. inundatus, P. lactucaedebilis, P. macrosporus, and P. pachydermus*) by its ability to utilize methyl-α-D-mannopyranoside. Morphologically differentiated based on the shorter (4.8 ± 1.6 μm) mean length of 3 d old yeast cells in culture on YPD medium compared to *P. inouyei* (6.3 ± 1.6 μm).

*Type*: **Finland**: Helsinki, 60.23270 ^o^N 25.06191 °E, isol, ex leaf wash of healthy wild growing thale cress (*Arabidopsis thaliana)*, May 2013, *K. Wang & K. Overmyer,* strain C29 (HAMBI 3697 – holotype preserved in a metabolically inactive state; DSM 110145 – ex-holotype culture). ITS sequence, Genebank LT602858.

**Fig. 8:**
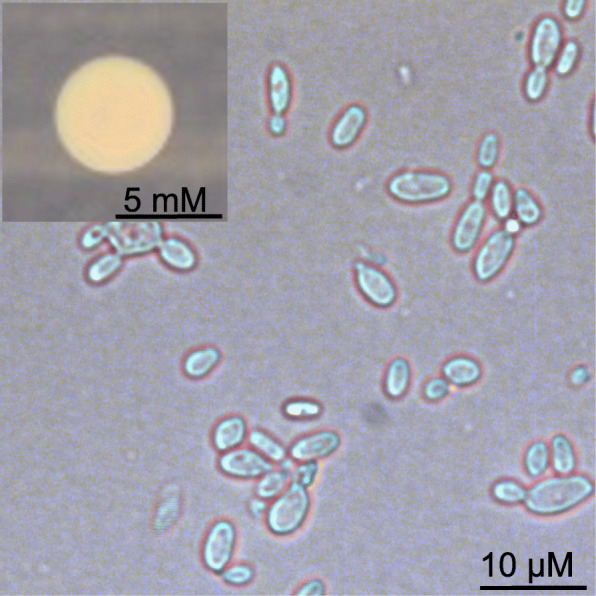
The morphology of *Protomyces arabidopsidicola* sp. nov. type strain C29. Photo plate depicts the micro-morphology of the yeast phase and the macro-morphology a colony solid agar plate (insert). In both photos cultivation was on YPG (yeast-extract, peptone, glucose) medium for 1 week (colony) and 3 days (yeast cells)

*Notes:* The genome size of *Protomyces arabidopsidicola* is 11.9 Mbp (50.9% GC content), with 5514 annotated protein-coding genes (Wang et al. 2019).

*Description: Haploid cells* yeast-like, oval 2.0 – 12.4 × 1.4 – 5.6 μm when cultured on YPG for 3 d at 21 °C (Fig. 8). *Single colonies* circular, convex, yellowish becoming slightly pinkish after about a week (Fig. 8, inset). *Growth* does not occur over 30 °C or below 8 °C on YPG agar medium for 7 d; slow growth with colony appearance at ≥ 2 weeks observed at 4 °C. *Carbon source utilization* Yeast-like cells can assimilate glycerol, D-xylose, D-glucose, D-fructose, D-mannose, D-mannitol, D-sorbitol, amygdalin, arbutin, esculin ferric citrate, salicin, D-cellobiose, D-maltose, D-saccharose (sucrose), D-trehalose, D-raffinose, amidon (starch), xylitol, gentiobiose, D-turanose, D-arabitol, and weakly growth with D-arabinose, L-arabinose, Methyl-α-D-mannopyranoside, Methyl-α-D-glucopranoside, D-melezitose, Potassium 2-ketogluconate, but do not assimilate erythritol, D-ribose, L-xylose, D-adonitol, Methyl-β-D-xylopyranoside, D-galactose, L-sorbose, L-rhamnose, dulcitol, inositol, N-acetylglucosamine, D-lactose, D-melibiose, inulin, glycogen, D-lyxose, D-tagatose, D-fucose, L-fucose, L-arabitol, potassium gluconate or potassium 5-ketogluconate.

*Additional material examined:* **Finland**: Helsinki, 60.23270 ^o^N 25.06191 °E, isol, ex leaf wash of healthy wild growing thale cress (*Arabidopsis thaliana)*, May 2013, *K. Wang & K. Overmyer* strains C2–11(HAMBI 3736 = DSM 111894) and C2–15 (HAMBI 3737 = DSM 111895).

## DISCUSSION

### Recognition of *Protomyces arabidopsidicola*

*P. arabidopsidicola* is recognized based on the phylogenetic data (Figs. 3, 4, and Wang et al. 2019) physiological characters (Fig. 2, Tables 2 and 4), its association with *Arabidopsis* (Agler et al. 2016; Wang et al. 2019), and the low ANI and AAI values between *P. arabidopsidicola* and other known *Protomyces* species (Table 4). This was isolated in an effort to establish experimental systems to study the genetics of plant/yeast interactions using the genetic model plant *Arabidopsis*, for which associated yeasts were previously few to unknown (Wang et al. 2016, 2019). We obtained three strains with identical ITS sequences, further justified by the strength of the whole genome phylogenetic data, and further the novelty of this species that interacts with a plant widely outside the known host range of other *Protomyces* species. The strength of the data supporting its association with *Arabidopsis* (Agler et al. 2016; Wang et al. 2016; Wang et al. 2019), demonstrates the need for further research on this grossly understudied genus.

Previous phylogenetic analysis of *Protomyces* species used the host tissue, in which ascogenous cells were formed, as characteristic for their classification. Further, morphology and cell size comparisons were typically done with both the ascogenous cells, which only exist during natural host infection, and cultured yeast phase cells. For reasons discussed below, *P. arabidopsidicola* strain C29 was not expected to cause disease. In the absence of infected host tissue, it was not possible to obtain ascogenous cells; thus only yeast cell sizes were compared to the six available *Protomyces* species. Our measurements of yeast cell sizes in the other *Protomyces* species (Table 2) were in agreement with previously published size ranges (Kurtzman 2011).

The application of molecular tools has extensively widened our knowledge of fungal diversity and phylogeny (Blackwell 2011; Crous et al. 2015; Rosling et al. 2011). Our results (Fig. 3 a-b) suggest that the true diversity of the genus *Protomyces* is not captured or supported in the D1/D2 or ITS phylogenies. Similar results were obtained in analyses using a different set of species in our previous work (Wang et al. 2019). Also, the same conclusion has been made previously in the sister genus *Taphrina* (Rodrigues and Fonseca 2003). Our full genome based phylogenetic tree resolved all species and was well supported (Fig. 3c), but is not practical for the rapid identification of *Protomyces* species. Five single gene nuclear markers, as used by Stielow et al. (2015), performed reasonably well at species resolution, and one marker, *act1,* exhibited the same architecture as the phylogenetic tree constructed with genome-wide concatenated protein data (Figs. 3c, 4). Further studies will be required to test how robust *act1* is in mimicking the topology of the genome wide tree when other species are added. However, as *act1* is a commonly used marker, we propose that once a new strain has been placed in *Protomyces* by ITS or D1/D2 sequencing, the *act1* gene sequence can be used as a secondary marker for species identification.

Carbon source utilization remains a useful tool for rapid species identification (Kurtzman et al. 2011), especially in a genus such as *Protomyces*, with a wealth of older literature and little molecular data available for comparison. Generally, the molecular underpinnings of carbon assimilation remain understudied. The availability of whole genome sequencing data offers an opportunity to correlate genomic carbon metabolism gene content with metabolic traits. Our data indicate that some traits correlate well with genomic content, while others do not (Fig. 5). A similar finding has been previously reported for D-xylose in a wider sampling of yeast species (Riley et al. 2016). The reasons for these differences remain unknown but this may account for discrepancies in carbon use traits observed between different labs or variable results seen within a single lab. Differences in the expression or conditional expression of carbon utilization genes may account for this; such latent metabolic capability has been previously suggested in a study using a wide selection of different yeasts of biotechnological interest (Riley et al. 2016).

### The association of *Protomyces* with *Arabidopsis thaliana*

*Arabidopsis* is very distantly related to the typical *Protomyces* hosts. Thus, we sought multiple lines of evidence to support the validity of the *Protomyces*-*Arabidopsis* association. In addition to our multiple isolations (Wang et al. 2016), *Protomyces* OTUs were also found over multiple years in two separate cities in Germany (Wang et al. 2019; Agler et al. 2016). Recently, *Protomyces*, or higher level phylogenetic classifications that include *Protomyces*, have been reported in other *Arabidopsis* phyllosphere microbiome studies (Brachi et al., 2017; Regalado et al. 2020).

All previously examined *Protomyces* species are heterothallic (Kurtzman 2011) and thus require conjugation with a partner of the opposite mating type prior to transitioning into the pathogenic hyphal form. *Protomyces* MAT loci analysis (Wang et al. 2019) confirmed heterothallism; all *Protomyces* species except one had a single MAT locus with either matPi/matPc or matMi/matMc. Exceptionally, *P. inundatus* had two MAT loci, one bearing matPi/matPc and the other matMi/matMc, suggesting homothallism in this species (Wang et al. 2019). As strain C29 has a single mat locus bearing only matPi/matPc it is not expected to be pathogenic without another strain bearing matMi/matMc. We confirmed the lack of pathogenicity in *Arabidopsis* infection experiments under a wide variety of conditions, including growth chamber experiments and overwinter field experiments, which revealed no disease symptoms (Wang et al. 2019). *P. arabidopsidicola* was then used to explore the role of phyllosphere residency in the *Protomyces* life-cycle. The species persisted in the *Arabidopsis* phylloplane of both sterile in vitro grown and soil grown plants, while titres of its closest relative *P. inouyei* rapidly decreased (Wang et al. 2019). Furthermore, *P. arabidopsidicola* was reisolated from *Arabidopsis* after overwintering for 6 months in field infection experiments (Wang et al. 2019). ITS metagenomics experiments have revealed *Protomyces* strains on the leaf surface of other plants that are not members of either *Compositae* or *Umbelliferae* (Wang et al. 2016; Prior et al. 2017; Wang et al. 2019). This suggests that *Protomyces* may exploit *Arabidopsis* and possibly also multiple other alternate hosts as a phyllosphere resident. We cannot at this time exclude the possibilities that *P. arabidopsidicola* is pathogenic on *Arabidopsis* or a different currently unknown host species. The collection of more strains of *Protomyces* from *Arabidopsis* and additional experimental evidence will be necessary for a deeper understanding of the ecology of the species.

Finally, comparative genomic analysis revealed the genomic signatures in *P. arabidopsidicola* consistent with a host jump or life-style change leading us to hypothesize that this fungus may have recently jumped hosts (Wang et al. 2019). Taken together, these data support the species being associated with *Arabidopsis*, whose phylloplane it can utilize as a growth space, as a possible host, or alternate host.

### *Protomyces* is not strictly related to hosts in *Compositae* or *Umbelliferae*

The genus *Protomyces* has been narrowly defined based on the following key criteria; morphological characteristics, host plant phylogeny, and their localization with the tissues of the host (Reddy and Kramer 1975; Kurtzman 2011). Currently all *Protomyces* species are known to be plant pathogens, infecting hosts in only two plant families. Previously, many yeasts have been excluded from *Protomyces* based on their atypical cell sizes and association with hosts in other plant families (Reddy and Kramer 1975). Our results placed *P. arabidopsidicola* as a district species within *Protomyces* and is associated with the *Arabidopsis* phylloplane, prompting us to propose the species name “*arabidopsidicola”*. These results indicate that the genus *Protomyces* may include species with a non-pathogenic phyllosphere resident life-style on alternate hosts and/or those associated with hosts outside *Umbelliferae* and *Compositae*, i.e. species that do not adhere to the narrow criteria previously used to define the genus. The isolation and characterization of *P. arabidopsidicola* as well as the identification of OTUs that belong to *Protomyces* based on their ITS sequence and reside on other plant families (Wang et al. 2019) both support this view. However, as stated above, we cannot at this time exclude the possibility that *P. arabidopsidicola* is pathogenic on a host that is currently unknown. Nonetheless, the occurrence of a *Protomyces* species in the phylloplane of a host species outside of the usual host range is novel. This suggests that *Protomyces* species may also survive via the utilization of the phylloplane of alternate hosts. Further studies will be required to fully resolve this issue, however, we propose here that the definition of the genus *Protomyces* be broadened to allow species with a phylloplane resident lifestyle and also species associated with hosts outside of *Umbelliferae* (*Apiaceae*) and *Compositae* (*Asteraceae*).

### Phylogenetic implications of *Protomyces* species genome sequencing

Phylogenomics with 636 conserved single copy concatenated nuclear encoded proteins confirmed the placement of *Protomyces* within *Taphrinomycotina* (Fig. 6, Supplemental Fig. 2)*.* However, our data place both *Saitoella* and *Neolecta* in a sister clade to that of *Taphrina* and *Protomyces* suggesting that *Saitoella* is outside the family *Protomycetaceae* and order *Taphrinales,* where it was previously assigned (Sugiyama et al. 2006). This suggests that *Saitoella* should either define its own family to be created and named or that this genus should be assigned to *Neolectales*. *Pneumocystis*, representative of the *Pneumocystidales*, was previously a sister group with *Schizosaccharomyces*, but our results now suggest it may reside as an outgroup between *Taphrinales* and *Schizosaccharomycetales*. Further studies with a wider selection of representative species will be required to better resolve the relationships within the subphylum *Taphrinomycotina*.

Our results and those of many others (Liu et al. 2009; Rosling et al. 2011) suggest there are a large number of undiscovered and lost species in *Taphrinomycotina,* whose discovery and analysis would aid in resolving the relationships in this fascinating subphylum. The family *Protomycetaceae* now contains the genera *Burenia*, *Protomyces*, *Protomycopsis*, *Saitoella*, *Taphridium*, and *Volkartia*. The borders between these genera also remain poorly defined and all but *Saitoella* have similar plant pathogenic life-styles. Unfortunately, with the exception of the genera *Protomyces* and *Saitoella*, strains and DNA sequences are not available for species in any of these genera.

Kurtzman (2011) concluded that his previous treatment of six *Protomyces* species, including *P. inouyei* and *P. lactucaedebilis,* could not conclude that all were distinct species. Our genomic data (Figs. 3, 4, 7, Tables 3, 4), suggest that *P. inouyei and P. lactucaedebilis* may be two strains of the same species. Phylogenies constructed with ITS and D1D2 sequences were either not well supported or did not resolve at the species level within the genus *Protomyces* (Fig. 3a-b). The ITS percent identity value between *P. inouyei* and *P. lactucaedebilis* was 98.3% (Table 3a), just under the 98.4% threshold for species delimitation (Vu et al. 2016). D1D2 percent identity values exceeded the 99.5% threshold for species delimitation (Vu et al. 2016) in several instances, including comparisons of clearly distinct species (Table 3b). The strongest evidence for merging *P. inouyei* and *P. lactucaedebilis* comes from whole genome phylogeny and ANI data (Fig. 3c; Table 4). Their ANI value exceeds the common border of 95% used for prokaryotes (Konstantinidis and Tiedje 2005; Goris et al. 2007). However, species delimitation borders using ANI and AAI values are not yet well established for yeasts and fungi, with limited data available. ANI values < 88% were reported between *Rhizoctonia solani* isolates from different anastomosis groups (Wibberg et al. 2015). In the same study, as a control ANI and AAI values were calculated for sequenced pairs of strains from five fungal species (*Aspergillus niger, Candida albicans*, *Crytococcus neoformans*, *Fusarium oxysporum*, and *Metarhizium anisopliae*), all of which were 97.42–99.97% for ANI and 98.11–99.98% for AAI. The ANI threshold of 95% in prokaryotes was originally justified based on its correspondence with the similarity index threshold of 70%, determined by DNA-DNA hybridization, a long-used standard for species delineation (Goris et al. 2007). A few fungal DNA-DNA hybridization studies have reported the same 70% similarity for *Fusarium* species (Kurtzman 1993b) suggesting the 95% ANI value may also be relevant to fungi. However, we contend that further studies are needed.

We conclude that *P. inouyei and P. lactucaedebilis* may be conspecific, but further studies into the use of ANI and AAI values for species delimitation are required before this can be formally proposed. Additionally, studies into the host specificities of these *Protomyces* species may also help resolve this issue. Although *P. inouyei* and *P. lactucaedebilis* are thought to have distinct hosts, infecting *Crepis* species and *Lactuca debilis*, respectively (Table 1), this has never been formally tested by reciprocal infections.

## CONCLUSIONS

The *Protomyces* strain C29 isolated from the phylloplane of *Arabidopsis* is confirmed as a new species named here as *P. arabidopsidicola*. Given the novel life-style and the association of this new species with a plant species outside of the previously accepted host range of the members of the genus *Protomyces*, we propose that the definition of the genus be widened to include non-pathogenic phylloplane-resident species and species associated with hosts outside families *Umbelliferae* (*Apiaceae*) and *Compositae* (*Asteraceae*).

## Supplementary Information

**Additional file 1: Supplemental Fig. 1.** Phylogenetic analysis of the genus *Protomyces*. Phylogenetic trees built by ITS (a), D1D2 (b) and genome-wide sequences (c), with Bayesian method. Bayesian phylogenetic trees were produced by using program MrBayes version 3.2.7a. The input .nex files are generated from the aligned fasta files used in (Fig. 1). General time reversible model and invgamma were chosen. Two independent analyses were started simultaneously, and 10,000 generations and four chains were set for analysis run. Posterior probability (%) support values are shown at each node. The output .tre files were viewed with online tool iTOL. In all phylogenies *Schizosaccharomyces pombe* was used as an outgroup. **Supplemental Fig. 2.** Phylogeny of the subphylum *Taphrinomycotina*. Maximum likelihood phylogenetic tree of representative species in the subphylum *Taphrinomycotina*. Trees were built using 636 single-copy protein sequences that were common to all species used. Alignment quality control of single-copy conserved proteins was achieved by applying sequence scores > = 0.8 in MAFFT analysis using Guidance2. *Saccharomyces cerevisiae* was used as an outgroup. Multiple aligned sequences of each species were concatenated into a single long sequence using FASconCAT_V1.0. Bayesian inference method results utilized the MrBayes software package. General time reversible model and invgamma were chosen. Two independent analyses were started simultaneously, and 10,000 generations and four chains were set for analysis run. Posterior probability (%) support values are shown at each node. The output files were viewed with online tool iTOL. **Supplemental file 1.** Queried yeast culture collections, the file contains a list of the thirty major yeast culture collections that were queried for availability of strains for species belonging to the genus *Protomyces.* **Supplemental file 2.** Carbon utilization enzyme protein sequences, the file contains the characterized protein sequences from model yeast species that were used as BLAST queries against the genomes of *Protomyces* species to identify genes involved in the utilization of various carbon sources.

## Data Availability

The datasets generated and/or analysed during the current study are available in the repositories listed below: Gene and genome sequences used here can be accessed in GenBank, www.ncbi.nlm.nih.gov/ genbank, under the following accession numbers: For ITS sequences: *Protomyces arabidopsidicola*, strain C29 (Para), LT602858; *P. gravidus* (Pgra), MK937055; *P. inouyei* (Pino), MK937056; *P. inundatus* (Pinu), MK937057; *P. lactucaedebilis* (Plac), MK937058; *P. macrosporus* (Pmac), MK937059; *P. pachydermus* (Ppac), MK937060. For D1/D2 sequences: Para, MK934482; Pgra, U84342.1; Pino, NG_042406.1; Pinu, U76528.1; Plac, U84343.1; Pmac, U94939; Ppac U84345. For act1: Para, MN031257; Pgra, MN031251; Pino, MN031252; Pinu, MN031253; Plac, MN031254; Pmac, MN031255; Ppac MN031256. For rbp1: Para, MN270968; Pgra, MN270962; Pino, MN270963; Pinu, MN270964; Plac, MN270965; Pmac, MN270966; Ppac MN270967. For rbp2: Para, MN313889; Pgra, MN313883; Pino, MN313884; Pinu, MN313885; Plac, MN313886; Pmac, MN313887; Ppac MN313888. For tef1: Para, MN304745; Pgra, MN304739; Pino, MN304740; Pinu, MN304741; Plac, MN304742; Pmac, MN304743; Ppac MN304744. For tub2: Para, MN178303; Pgra, MN178297; Pino, MN178298; Pinu, MN178299; Plac, MN178300; Pmac, MN178301; Ppac, MN178302. The GenBank accession number of *Protomyces arabidopsidicola* strain C29 genome is QXMI00000000 and genome raw data in SRA is SRR8109439. Genome annotations available at genomevolution.org/coge/GenomeInfo.pl? with the following genome IDs: Para, 53653; Pgra, 53651; Pino, 53654; Pinu, 53676; Plac, 54947; Pmac, 53670; Ppac 54948. The *Protomyces arabidopsidicola* strain C29 has been deposited as the holotype in the University of Helsinki Microbial Domain Biological Resource Centre (HAMBI) Culture Collection, www.helsinki.fi/en/infrastructures/biodiversity-collections/infrastructures/microbial-domain-biological-resource-centre-hambi, under the accession no. HAMBI3697, and an ex-holotype culture was deposited in the German Collection of Microorganisms and Cell Cultures (DSMZ) culture collection, www.dsmz.de, under the accession no. DSM 110145. The strains SC2–11 and SC2–15 are available from the same collections under the following accession numbers; strains C2–11, HAMBI 3736, DSM 111894, and C2–15,HAMBI 3737, DSM 111895. The species name *Protomyces arabidopsidicola* has been registered with Mycobank, http://www.mycobank.org/, under the accession no. MB 830646.

## Abbreviations

nrDNA: Nuclear ribosomal DNA
D1/D2: nrDNA 26S large-subunit D1/D2 domain
ITS: nrDNA internal transcribed spacer
ANI: Average nucleotide similarity
AAI: Average amino acid similarity
YPG: Yeast-extract, peptone, glucose medium
*act1*: Actin1 gene
*rbp2*: Gene for the second largest subunit of RNA polymerase II
*rbp1*: Gene for the large subunit of RNA polymerase II
*tef1*: Transcription elongation factor 1 gene
*tub2*: Tubulin beta chain gene
OTU: Operational taxonomic unit
HAMBI: University of Helsinki HAMBI Culture Collection
DSM: German Collection of Microorganisms and Cell Cultures GmbH
Para: *Protomyces arabidopsidicola*
Pgra: *P. gravidus*
Pino: *P. inouyei*
Pinu: *P. inundatus*
Plac: *P. lactucaedebilis*
Pmac: *P. macrosporus*
Ppac: *P. pachydermus*
