# Supplementary material for: A novel Arabidopsis phyllosphere resident Protomyces species and a re-examination of genus Protomyces based on genome sequence data
Source: IMA Fungus. 2021 Mar 19;12:8. doi: 10.1186/s43008-021-00054-2 (PMC7980564; doi:10.1186/s43008-021-00054-2)
Supplement: Supplementary file 1 — Additional file 1: Supplemental Fig. 1. Phylogenetic analysis of the genus Protomyces. Phylogenetic trees built by ITS (a), D1D2 (b) and genome-wide sequences (c), with Bayesian method. Bayesian phylogenetic trees were produced by using program MrBayes version 3.2.7a. The input .nex files are generated from the aligned fasta files used in (Fig. 1). General time reversible model and invgamma were chosen. Two independent analyses were started simultaneously, and 10,000 generations and four chains were set for analysis run. Posterior probability (%) support values are shown at each node. The output .tre files were viewed with online tool iTOL. In all phylogenies Schizosaccharomyces pombe was used as an outgroup. Supplemental Fig. 2. Phylogeny of the subphylum Taphrinomycotina. Maximum likelihood phylogenetic tree of representative species in the subphylum Taphrinomycotina. Trees were built using 636 single-copy protein sequences that were common to all species used. Alignment quality control of single-copy conserved proteins was achieved by applying sequence scores > = 0.8 in MAFFT analysis using Guidance2. Saccharomyces cerevisiae was used as an outgroup. Multiple aligned sequences of each species were concatenated into a single long sequence using FASconCAT_V1.0. Bayesian inference method results utilized the MrBayes software package. General time reversible model and invgamma were chosen. Two independent analyses were started simultaneously, and 10,000 generations and four chains were set for analysis run. Posterior probability (%) support values are shown at each node. The output files were viewed with online tool iTOL. Supplemental file 1. Queried yeast culture collections, the file contains a list of the thirty major yeast culture collections that were queried for availability of strains for species belonging to the genus Protomyces. Supplemental file 2. Carbon utilization enzyme protein sequences, the file contains the characterized protein sequences from model [file 43008_2021_54_MOESM1_ESM.pdf]

Supplemental Fig. 1

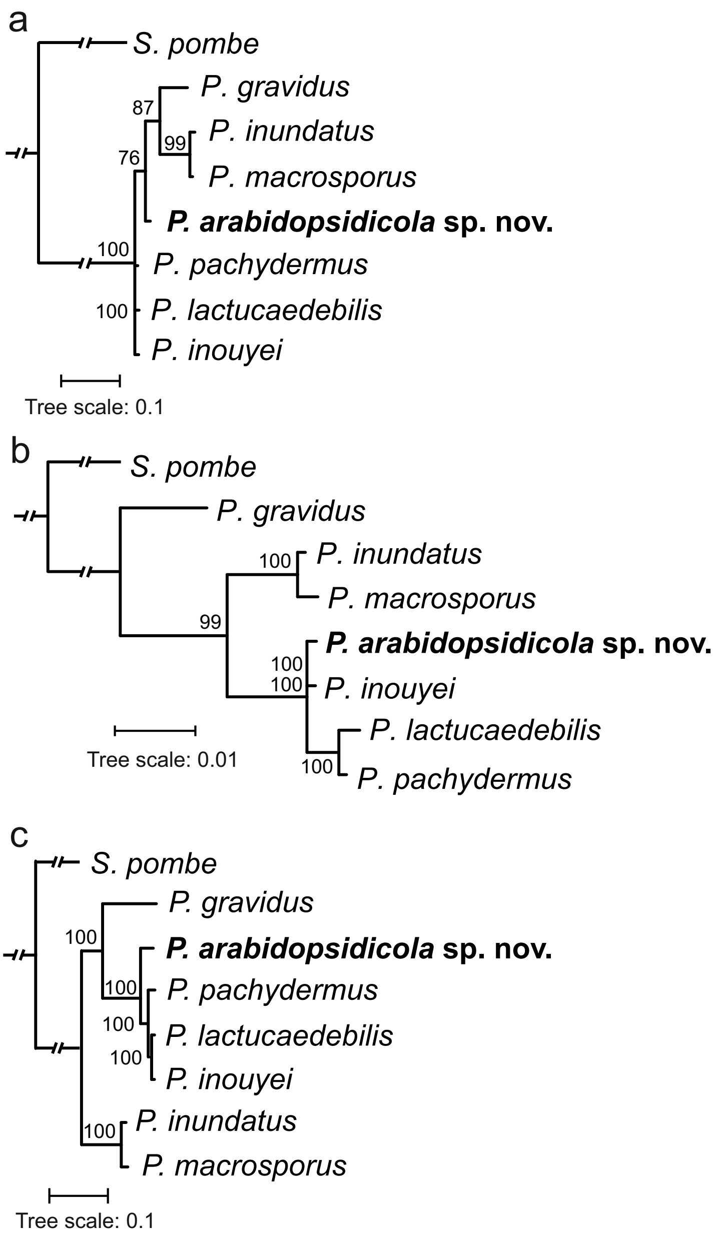

Supplemental Fig. 2

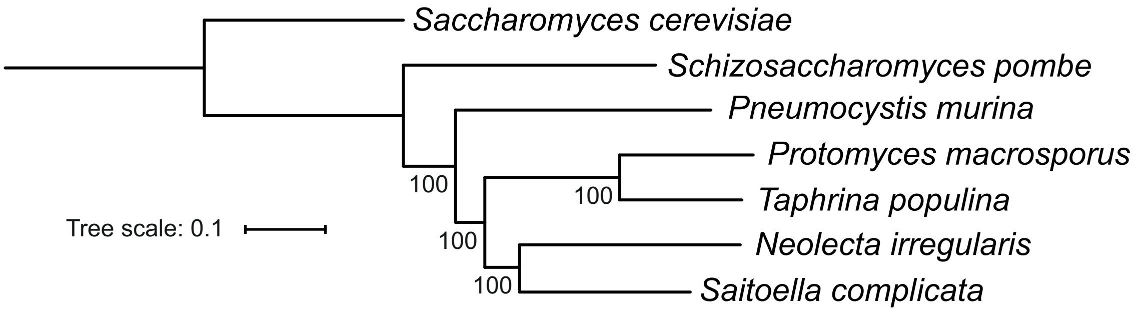

Supplemental file 1. Yeast culture collections queried.

List of thirty major yeast culture collections that were queried for availability of strains of species belonging to the genus *Protomyces*. Search was done in in July 2019. The queried collections were:

The China General Microbiological Culture Collection Center, Beijing, China (CGMCC; http://www.cgmcc.net/english)

Microbial Domain Biological Resource Centre, Helsinki, Finland (HAMBI; https://www.helsinki.fi/en/infrastructures/biodiversity-collections/infrastructures/microbial-domain-biological-resource-centre-hambi).

VTT Technical Research Center of Finland Culture Collection, Espoo, Finland (http://culturecollection.vtt.fi/m).

The German Collection of Microorganisms and Cell Cultures GmbH, Braunschweig, Germany (DSMZ; www.dsmz.de).

The Industrial Yeasts Collection, Perugia, Italy (DBVPG; http://www.dbvpg.unipg.it/index.php/en).

The Japan Collection of Microorganisms, Koyadai, Tsukuba, Ibaraki, Japan (JCM; jcm.brc.riken.jp/en/).

Biological Resource Center NITE, Chiba, Japan (NBRC; www.nite.go.jp/en/nbrc).

CBS-KNAW Fungal Biodiversity Centre, Utrecht, The Netherlands (CBS; http://www.westerdijkinstitute.nl/Collections/).

The Portuguese Yeast Culture Collection, Caparica, Portugal (PYCC; http://pycc.bio-aware.com/).

The All-Russian Collection of Microorganisms, Pushchino and Moskow Russia (VKM; http://www.vkm.ru).

Culture Collection of Yeasts, Bratislava, Slovakia (CCY; http://ccy.sk/index.php/en/).

National Collections of Yeast Cultures, Norwich, UK (NCYC; www.ncyc.co.uk).

Agricultural Research Service Culture Collection, Peoria, IL, USA (NRRL; nrrl.ncaur.usda.gov).

American Type Culture Collection, Manassas, VA, USA (ATCC; www.atcc.org).

University of California Phaff Culture Collection, Davis, CA, USA (UCD-FST; http://phaffcollection.ucdavis.edu).

Belgian Coordinated Collection of Microorganisms, Louvain-la-Neuve, Belgium (BCCM/MCLU; http://bccm.belspo.be/).

Bioresource Collection and Research Center, Hsinchu, Taiwan (BCRC; catalog.bcrc.firdi.org.tw).

Chinese Center for Industrial Culture Collection, Beijing, China (CICC; http://english.china-cicc.org/).

Centre International de Ressources Microbiennes-Levures, Levure, France (CIRM-Levures; https://www6.inra.fr/cirm_eng/Yeasts).

Spanish Type Culture Collection, Valencia, Spain (CECT; https://www.uv.es/cect).

Korean Collection for Type Cultures, Jeollabuk-do, Korea (KCTC; https://kctc.kribb.re.kr/En/Kctc.aspx).

ZIM Collection of Industrial Microorganisms, University of Ljubljana, Ljubljana, Slovenia (ZIM; www.bf.uni-lj.si/zt/biotech/chair/index.html).

Universidade Federal de Pernambuco, Micoteca do Departmento de Micologia, Recife, Brazil (URM; https://www.ufpe.br/micoteca/).

Lallemand Yeast Culture Collection, Lallemand Inc., Canada (LYCC; www.lallemand.com/).

National Collection of Agricultural and Industrial Microorganisms, Budapest, Hungary (NCAIM; ncaim.etk.szie.hu/).

National Bank for Industrial Microorganisms and Cell Cultures, University of Chemical Technology and Metallurgy, Sofia, Bulgaria (NBIMCC; www.nbimcc.org).

Microbial Type Culture Collection, Institute of Microbial Technology, Chandigarh, India (MTCC; https://mtccindia.res.in/).

Collection of Industrial Microorganisms, Institute of Agricultural and Food Biotechnology, Warsaw, Poland (IAFB; https://cim.ibprs.pl/).

Food Science Australia, Ryde, CSIRO, North Ryde, Australia (FRR; www.foodscience.csiro.au/fcc/services.htm).

The UAMH Centre for Global Microfungal Biodiversity, Toronto, Canada (UAMH; www.uamh.ca).

Supplemental file 2. Carbon utilization enzyme protein sequences.

Characterized protein sequences from model yeast species that were used as BLAST queries against the genomes of *Protomyces* species to identify genes involved in the utilization of various carbon sources.

>sp|P25553|ALDA_ECOLI Lactaldehyde dehydrogenase OS=Escherichia coli (strain K12) GN=aldA PE=1 SV=2

MSVPVQHPMYIDGQFVTWRGDAWIDVVNPATEAVISRIPDGQAEDARKAIDAAERAQPEWE
ALPAIERASWLRKISAGIRERASEISALIVEEGGKIQQLAEVEVAFTADYIDYMAEWARRYEGEI
IQSDRPGENILLFKRALGVTTGILPWNFPFFLIARKMAPALLTGNTIVIKPSEFTPNNAIAFAKIV
DEIGLPRGVFNLVLGRGETVGQELAGNPKVAMVSMTGSVSAGEKIMATAAKNITKVCLELGG
KAPAIVMDDADLELAVKAIVDSRVINSGQVCNCAERVYVQKGIYDQFVNRLGEAMQAVQFGN
PAERNDIAMGPLINAAALERVEQKVARAVEEGARVAFGGKAVEGKGYYYPPTLLLDVRQEM
SIMHEETFGPVLPVVAFDTLEDAISMANDSDYGLTSSIYTQNLNVAMKAIKGLKFGETYINREN
FEAMQGFHAGWRKSGIGGADGKHGLHEYLQTQVVYLQS

>sp|Q97U96|ARAD_SULSO Arabinonate dehydratase OS=Sulfolobus solfataricus (strain ATCC 35092 / DSM 1617 / JCM 11322 / P2) GN=araD PE=1 SV=1

MIKDIRTYKLCYEGINDERDALAIKGLAEHPMEIVATEIETSDGYVGYGESLAYGCSDAVQVTI
EKILKPLLLKEDEELIEYLWDKMYKATLRFGRRGIAIAGISGVDTALWDIMGKKAKKPIYKLLGG
SKRKVRAYITGGYYSEKKDLEKLRDEEAYYVKMGFKGIKVKIGAKSMEEDIERLKAIREVVGE
DVKIAVDANNVYTFEEALEMGRRLEKLGIWFFEEPIQTDYLDLSARLAEELEVPIAGYETAYTR
WEFYEIMRKRAVDIVQTDVMWTGGISEMMKIGNMAKVMGYPLIPHYSAGGISLIGNLHVAAA
LNSPWIEMHLRKNDLRDKIFKESIEIDNGHLVVPDRPGLGYTIRDGVFEEYKCKS

>sp|Q97UA1|KGSDH_SULSO 2,5-dioxopentanoate dehydrogenase OS=Sulfolobus solfataricus (strain ATCC 35092 / DSM 1617 / JCM 11322 / P2) GN=aldhT PE=1 SV=1

MKSYQGLADKWIKGSGEEYLDINPADKDHVLAKIRLYTKDDVKEAINKAVAKFDEWSRTPAP
KRGSILLKAGELMEQEAQEFALLMTLEEGKTLKDSMFEVTRSYNLLKFYGALAFKISGKTLPS
ADPNTRIFTVKEPLGVVALITPWNFPLSIPVWKLAPALAAGNTAVIKPATKTPLMVAKLVEVLS
KAGLPEGVVNLVVGKGSEVGDTIVSDDNIAAVSFTGSTEVGKRIYKLVGNKNRMTRIQLELG
GKNALYVDKSADLTLAAELAVRGGFGLTGQSCTATSRLIINKDVYTQFKQRLLERVKKWRVG
PGTEDVDMGPVVDEGQFKKDLEYIEYGKNVGAKLIYGGNIIPGKGYFLEPTIFEGVTSDMRLF
KEEIFGPVLSVTEAKDLDEAIRLVNAVDYGHTAGIVASDIKAINEFVSRVEAGVIKVNKPTVGLE
LQAPFGGFKNSGATTWKEMGEDALEFYLKEKTVYEGW

>sp|P0AB87|FUCA_ECOLI L-fuculose phosphate aldolase OS=Escherichia coli (strain K12) GN=fucA PE=1 SV=1

MERNKLARQIIDTCLEMTRLGLNQGTAGNVSVRYQDGMLITPTGIPYEKLTESHIVFIDGNGK
HEEGKLPSSEWRFHMAAYQSRPDANAVVHNHAVHCTAVSILNRSIPAIHYMIAAAGGNSIPC
APYATFGTRELSEHVALALKNRKATLLQHHGLIACEVNLEKALWLAHEVEVLAQLYLTTLAITD
PVPVLSDEEIAVVLEKFKTYGLRIEE

>sp|P11553|FUCK_ECOLI L-fuculokinase OS=Escherichia coli (strain K12) GN=fucK PE=1 SV=3

MLSGYIAGAIMKQEVILVLDCGATNVRAIAVNRQGKIVARASTPNASDIAMENNTWHQWSLD
AILQRFADCCRQINSELTECHIRGIAVTTFGVDGALVDKQGNLLYPIISWKCPRTAAVMDNIER
LISAQRLQAISGVGAFSFNTLYKLVWLKENHPQLLERAHAWLFISSLINHRLTGEFTTDITMAG
TSQMLDIQQRDFSPQILQATGIPRRLFPRLVEAGEQIGTLQNSAAAMLGLPVGIPVISAGHDT
QFALFGAGAEQNEPVLSSGTWEILMVRSAQVDTSLLSQYAGSTCELDSQAGLYNPGMQWL
ASGVLEWVRKLFWTAETPWQMLIEEARLIAPGADGVKMQCDLLSCQNAGWQGVTLNTTRG
HFYRAALEGLTAQLQRNLQMLEKIGHFKASELLLVGGGSRNTLWNQIKANMLDIPVKVLDDA
ETTVAGAALFGWYGVGEFNSPEEARAQIHYQYRYFYPQTEPEFIEEV

>sp|P69922|FUCI_ECOLI L-fucose isomerase OS=Escherichia coli (strain K12) GN=fucI PE=1 SV=1

MKKISLPKIGIRPVIDGRRMGVRESLEEQTMNMAKATAALLTEKLRHACGAAVECVISDTCIA
GMAEAAACEEKFSSQNVGLTITVTPCWCYGSETIDMDPTRPKAIWGFNGTERPGAVYLAAA
LAAHSQKGIPAFSIYGHDVQDADDTSIPADVEEKLLRFARAGLAVASMKGKSYLSLGGVSMGI
AGSIVDHNFFESWLGMKVQAVDMTELRRRIDQKIYDEAELEMALAWADKNFRYGEDENNKQ
YQRNAEQSRAVLRESLLMAMCIRDMMQGNSKLADIGRVEESLGYNAIAAGFQGQRHWTDQ
YPNGDTAEAILNSSFDWNGVREPFVVATENDSLNGVAMLMGHQLTGTAQVFADVRTYWSP
EAIERVTGHKLDGLAEHGIIHLINSGSAALDGSCKQRDSEGNPTMKPHWEISQQEADACLAA
TEWCPAIHEYFRGGGYSSRFLTEGGVPFTMTRVNIIKGLGPVLQIAEGWSVELPKDVHDILNK
RTNSTWPTTWFAPRLTGKGPFTDVYSVMANWGANHGVLTIGHVGADFITLASMLRIPVCMH
NVEETKVYRPSAWAAHGMDIEGQDYRACQNYGPLYKR

>sp|Q97UA0|KDAD_SULSO 2-dehydro-3-deoxy-D-arabinonate dehydratase OS=Sulfolobus solfataricus (strain ATCC 35092 / DSM 1617 / JCM 11322 / P2) GN=kdaD PE=1 SV=1

MHFIMMKLFRVVKRGYYISYAILDNSTIIRLDEDPIKALMRYSENKEVLGDRVTGIDYQSLLKSF
QINDIRITKPIDPPEVWGSGISYEMARERYSEENVAKILGKTIYEKVYDAVRPEIFFKATPNRCV
GHGEAIAVRSDSEWTLPEPELAVVLDSNGKILGYTIMDDVSARDLEAENPLYLPQSKIYAGCC
AFGPVIVTSDEIKNPYSLDITLKIVREGRVFFEGSVNTNKMRRKIEEQIQYLIRDNPIPDGTILTT
GTAIVPGRDKGLKDEDIVEITISNIGTLITPVKKRRKIT

>sp|Q04585|YDR09_YEAST Uncharacterized sugar kinase YDR109C OS=Saccharomyces cerevisiae (strain ATCC 204508 / S288c) GN=YDR109C PE=1 SV=1

MKSRKRQNNMQNETREPAVLSSQETSISRISPQDPEAKFYVGVDVGTGSARACVIDQSGNM
LSLAEKPIKREQLISNFITQSSREIWNAVCYCVRTVVEESGVDPERVRGIGFDATCSLVVVSAT
NFEEIAVGPDFTNNDQNIILWMDHRAMKETEEINSSGDKCLKYVGGQMSVEMEIPKIKWLKN
NLEAGIFQDCKFFDLPDYLTFKATGKENRSFCSAVCKQGFLPVGVEGSDIGWSKEFLNSIGL
SELTKNDFERLGGSLREKKNFLTAGECISPLDKKAACQLGLTEHCVVSSGIIDAYAGWVGTVA
AKPESAVKGLAETENYKKDFNGAIGRLAAVAGTSTCHILLSKNPIFVHGVWGPYRDVLARGF
WAAEGGQSCTGVLLDHLITTHPAFTELSHMANLAGVSKFEYLNKILETLVEKRKVRSVISLAK
HLFFYGDYHGNRSPIADPNMRACIIGQSMDNSIEDLAVMYLSACEFISQQTRQIIEVMLKSGH

EINAIFMSGGQCRNSLLMRLLADCTGLPIVIPRYVDAAVVFGSALLGAAASEDFDYTREKRTL
KGQKSSQTKTERFNDSYSSIQKLSMEDRNSTNGFVSPHNLQLSTPSAPAKINNYSLPICTQQ
PLDKTSEESSKDASLTVGQESLGEGRYNGTSFLWKVMQELTGNARIVNPNEKTHPDRILLDT
KYQIFLDMIETQRKYRRMVDKVEGSFSR

>tr|Q9I1Q0|Q9I1Q0_PSEAE Probable aldehyde dehydrogenase OS=Pseudomonas aeruginosa (strain ATCC 15692 / DSM 22644 / CIP 104116 / JCM 14847 / LMG 12228 / 1C / PRS 101 / PAO1) GN=PA2217 PE=4 SV=1

MTAILGHNFIGGARSAAGTLFLQSLDAASGEALPYRFVQATPEEVDAAAEAAASAYPHYRQL
PASRRAEFLDTIAAELDALDDDFVAIVCRETALPATRIQGERSRTSGQLRLFAEVLRRGDFHG
ARIDRARPERKPLPRVDLRQCRIGLGPVAVFGASNFPLAFSTAGGDTAAALAAGCPVVFKAH
SGHMATAERVAAAILRAAERTGMPAGVFNMIYGGGVGERLVRHPAIQAVGFTGSLKGGRAL
CDMAAARAQPIPVFAEMSSINPVVLLPAALKKRGEAVADELSASVVLGCGQFCTNPGLVIGIR
SAQFSAFLERFAARMDDQPAQTMLNTGTLASYEKGLAALHAHPRVRHLAGQPQEGRQARP
QLFQADVSLLLEGDELLQEEVFGPASVVVEVADHAELKRALHGLHGQLTATLIAEAEDLASFA
DLVPLLEQKAGRLLLNGYPTGVEVCDAMVHGGPYPATSDARGTSVGTLAIDRFLRPVCYQN
YPDAWLPEALKDGNPLGIARLVDGIVTRAAVA

>Ss-araDH_tr|Q97YM2|Q97YM2_SULSO Alcohol dehydrogenase (Zn containing) (Adh-4) OS=Sulfolobus solfataricus (strain ATCC 35092 / DSM 1617 / JCM 11322 / P2) GN=adh-4 PE=1 SV=1

MENVNMVKSKAALLKKFSEPLSIEDVNIPEPQGEEVLIRIGGAGVCRTDLRVWKGVEAKQGF
RLPIILGHENAGTIVEVGELAKVKKGDNVVVYATWGDLTCRYCREGKFNICKNQIIPGQTTNG
GFSEYMLVKSSRWLVKLNSLSPVEAAPLADAGTTSMGAIRQALPFISKFAEPVVIVNGIGGLA
VYTIQILKALMKNITIVGISRSKKHRDFALELGADYVSEMKDAESLINKLTDGLGASIAIDLVGTE
ETTYNLGKLLAQEGAIILVGMEGKRVSLEAFDTAVWNKKLLGSNYGSLNDLEDVVRLSESGKI
KPYIIKVPLDDINKAFTNLDEGRVDGRQVITP

>tr|Q88NF5|Q88NF5_PSEPK Putative alpha-ketoglutarate semialdehyde dehydrogenase OS=Pseudomonas putida (strain ATCC 47054 / DSM 6125 / NCIMB 11950 / KT2440) GN=PP_1256 PE=4 SV=1

MPLTGNLLIGQRPVTGSRDAIRAIDPTTGQTLEPAYLGGTGEHVAQACALAWAAFDAYRETS
LEQRAEFLEAIATQIEALGDALIDRAVIETGLPKARIQGERGRTCTQLRTFARTVRAGEWLDVR
IDSALPERQPLPRADLRQRQVALGPVAVFGASNFPLAFSVAGGDTASALAAGCPVVVKAHSA
HPGTSELVGQAVAQAVKQCGLPEGVFSLLYGSGREVGIALVSDPRIKAVGFTGSRSGGMAL
CQAAQARPEPIPVYAEMSSINPVFLFDAALQARAEALAQGFVASLTQGAGQFCTNPGLVIAR
QGPALQRFITAAAGYVQQGAAQTMLTPGIFSAYQAGIAALADNPHAQAITSGQAGQGPNQC
QAQLFVTQAEAFLADPALQAEVFGAASLVVACTDDEQVRQVAEHLEGQLTATLQLDEADIDS
ARALLPTLERKAGRILVNGWPTGVEVCDAMVHGGPFPATSDARTTSVGTAAILRFLRPVCYQ
DVPDALLPQALKHGNPLQLRRLLDGKRED

>sp|P08204|ARAB_ECOLI Ribulokinase OS=Escherichia coli (strain K12) GN=araB PE=1 SV=4

MAIAIGLDFGSDSVRALAVDCATGEEIATSVEWYPRWQKGQFCDAPNNQFRHHPRDYIESM
EAALKTVLAELSVEQRAAVVGIGVDSTGSTPAPIDADGNVLALRPEFAENPNAMFVLWKDHT
AVEEAEEITRLCHAPGNVDYSRYIGGIYSSEWFWAKILHVTRQDSAVAQSAASWIELCDWVP
ALLSGTTRPQDIRRGRCSAGHKSLWHESWGGLPPASFFDELDPILNRHLPSPLFTDTWTADI
PVGTLCPEWAQRLGLPESVVISGGAFDCHMGAVGAGAQPNALVKVIGTSTCDILIADKQSVG
ERAVKGICGQVDGSVVPGFIGLEAGQSAFGDIYAWFGRVLGWPLEQLAAQHPELKTQINAS
QKQLLPALTEAWAKNPSLDHLPVVLDWFNGRRTPNANQRLKGVITDLNLATDAPLLFGGLIA
ATAFGARAIMECFTDQGIAVNNVMALGGIARKNQVIMQACCDVLNRPLQIVASDQCCALGAAI
FAAVAAKVHADIPSAQQKMASAVEKTLQPCSEQAQRFEQLYRRYQQWAMSAEQHYLPTSA
PAQAAQAVATL

>sp|P08202|ARAA_ECOLI L-arabinose isomerase OS=Escherichia coli (strain K12) GN=araA PE=1 SV=3

MTIFDNYEVWFVIGSQHLYGPETLRQVTQHAEHVVNALNTEAKLPCKLVLKPLGTTPDEITAI
CRDANYDDRCAGLVVWLHTFSPAKMWINGLTMLNKPLLQFHTQFNAALPWDSIDMDFMNL
NQTAHGGREFGFIGARMRQQHAVVTGHWQDKQAHERIGSWMRQAVSKQDTRHLKVCRFG
DNMREVAVTDGDKVAAQIKFGFSVNTWAVGDLVQVVNSISDGDVNALVDEYESCYTMTPAT
QIHGKKRQNVLEAARIELGMKRFLEQGGFHAFTTTFEDLHGLKQLPGLAVQRLMQQGYGFA
GEGDWKTAALLRIMKVMSTGLQGGTSFMEDYTYHFEKGNDLVLGSHMLEVCPSIAAEEKPIL
DVQHLGIGGKDDPARLIFNTQTGPAIVASLIDLGDRYRLLVNCIDTVKTPHSLPKLPVANALWK
AQPDLPTASEAWILAGGAHHTVFSHALNLNDMRQFAEMHDIEITVIDNDTRLPAFKDALRWN
EVYYGFRR

>sp|P08203|ARAD_ECOLI L-ribulose-5-phosphate 4-epimerase AraD OS=Escherichia coli (strain K12) GN=araD PE=1 SV=2

MLEDLKRQVLEANLALPKHNLVTLTWGNVSAVDRERGVFVIKPSGVDYSVMTADDMVVVSIE
TGEVVEGTKKPSSDTPTHRLLYQAFPSIGGIVHTHSRHATIWAQAGQSIPATGTTHADYFYGT
IPCTRKMTDAEINGEYEWETGNVIVETFEKQGIDAAQMPGVLVHSHGPFAWGKNAEDAVHN
AIVLEEVAYMGIFCRQLAPQLPDMQQTLLDKHYLRKHGAKAYYGQ

>sp|Q91XV4|DCXR_MESAU L-xylulose reductase OS=Mesocricetus auratus GN=DCXR PE=1 SV=1

MDLGLAGRRALVTGAGKGIGRSTVLALQAAGAHVVAVSRTQADLDSLVSECPGVETVCVDL
ADWEATEQALSSVGPVDLLVNNAAVALLQPFLEVTKEAFDMSFNVNLRAVIQVSQIVARGMI
ARGAPGAIVNVSSQASQRALANHSVYCSTKGALDMLTKMMALELGPHKIRVNAVNPTVVMT
SMGRTNWSDPHKAKVMLDRIPLGKFAEVENVVDAILFLLSHRSNMTTGSTLPVDGGFLVT

>sp|A2QAC0|LAD_ASPNC L-arabinitol 4-dehydrogenase OS=Aspergillus niger (strain CBS 513.88 / FGSC A1513) GN=ladA PE=1 SV=1

MATATVLEKANIGVFTNTKHDLWVADAKPTLEEVKNGQGLQPGEVTIEVRSTGICGSDVHFW
HAGCIGPMIVTGDHILGHESAGQVVAVAPDVTSLKPGDRVAVEPNIICNACEPCLTGRYNGC
ENVQFLSTPPVDGLLRRYVNHPAIWCHKIGDMSYEDGALLEPLSVSLAGIERSGLRLGDPCL
VTGAGPIGLITLLSARAAGASPIVITDIDEGRLEFAKSLVPDVRTYKVQIGLSAEQNAEGIINVFN

DGQGSGPGALRPRIAMECTGVESSVASAIWSVKFGGKVFVIGVGKNEMTVPFMRLSTWEID
LQYQYRYCNTWPRAIRLVRNGVIDLKKLVTHRFLLEDAIKAFETAANPKTGAIKVQIMSSEDDV
KAASAGQKI

>sp|A0QXD8|ELTD_MYCS2 Erythritol/L-threitol dehydrogenase OS=Mycobacterium smegmatis (strain ATCC 700084 / mc(2)155) GN=eltD PE=1 SV=1

MSNQVPEKMQAVVCHGPHDYRLEEVAVPQRKPGEALIRVEAVGICASDLKCYHGAAKFWG
DENRPAWAETMVIPGHEFVGRVVELDDEAAQRWGIAVGDRVVSEQIVPCWECLFCKRGQY
HMCQPHDLYGFKRRTPGAMASYMVYPAEALVHKVSPDIPAQHAAFAEPLSCSLHAVERAQI
TFEDTVVVAGCGPIGLGMIAGAKAKSPMRVIALDMAPDKLKLAEKCGADLTINIAEQDAEKIIK
DLTGGYGADVYIEGTGHTSAVPQGLNLLRKLGRYVEYGVFGSDVTVDWSIISDDKELDVLGA
HLGPYCWPAAIKMIESGALPMDEICTHQFPLTEFQKGLDLVASGKESVKVSLIPA

>sp|O93715|GLCDH_SULSF Glucose 1-dehydrogenase OS=Sulfolobus solfataricus GN=gdh PE=1 SV=1

MKAIIVKPPNAGVQVKDVDEKKLDSYGKIKIRTIYNGICGTDREIVNGKLTLSTLPKGKDFLVLG
HEAIGVVEESYHGFSQGDLVMPVNRRGCGICRNCLVGRPDFCETGEFGEAGIHKMDGFMR
EWWYDDPKYLVKIPKSIEDIGILAQPLADIEKSIEEILEVQKRVPVWTCDDGTLNCRKVLVVGT
GPIGVLFTLLFRTYGLEVWMANRREPTEVEQTVIEETKTNYYNSSNGYDKLKDSVGKFDVIID
ATGADVNILGNVIPLLGRNGVLGLFGFSTSGSVPLDYKTLQEIVHTNKTIIGLVNGQKPHFQQA
VVHLASWKTLYPKAAKMLITKTVSINDEKELLKVLREKEHGEIKIRILWE

>sp|Q96V44|LAD_HYPJE L-arabinitol 4-dehydrogenase OS=Hypocrea jecorina GN=lad1 PE=1 SV=1

MSPSAVDDAPKATGAAISVKPNIGVFTNPKHDLWISEAEPSADAVKSGADLKPGEVTIAVRST
GICGSDVHFWHAGCIGPMIVEGDHILGHESAGEVIAVHPTVSSLQIGDRVAIEPNIICNACEPC
LTGRYNGCEKVEFLSTPPVPGLLRRYVNHPAVWCHKIGNMSWENGALLEPLSVALAGMQR
AKVQLGDPVLVCGAGPIGLVSMLCAAAAGACPLVITDISESRLAFAKEICPRVTTHRIEIGKSA
EETAKSIVSSFGGVEPAVTLECTGVESSIAAAIWASKFGGKVFVIGVGKNEISIPFMRASVREV
DIQLQYRYSNTWPRAIRLIESGVIDLSKFVTHRFPLEDAVKAFETSADPKSGAIKVMIQSLD

>sp|G0RH19|LXR3_HYPJQ L-xylulose reductase OS=Hypocrea jecorina (strain QM6a) GN=lxr3 PE=1 SV=1

MKNGAFPHDNAAVPNVERVLPLFSLKGRTAIVSGAGAGIGLAVAQAFAEAGANVAIWYNSNK
QAVTSAEDIAKTYGVKCKAYQVNVTSAEAVDKAITEIIKEFNGRLDVFVANSGITWTEGAFIDG
SVESARNVMSVNVDGVMWCAKSAGAHFRRQKEEGTTIDGKPLDNFIAGSFIATASMSGSIV
NVPQLQAVYNSSKAAVIHFCKSLAVEWTGFARVNTVSPGYIITEISNFVPPETKTLWKDKIVM
GREGRVGELKGAYLYLASDASSYTTGLDMIVDGGYSLP

>sp|Q876L8|XYL1_HYPJE NAD(P)H-dependent D-xylose reductase xyl1 OS=Hypocrea jecorina GN=xyl1 PE=1 SV=1

MASPTLKLNSGYDMPQVGFGLWKVDNAVCADTVYNAIKAGYRLFDGACDYGNEKECGEGV
ARAIKDGLVKREDLFIVSKLWQTFHDEDKVEPITRRQLADWQIDYFDLFLVHFPAALEYVDPS

VRYPPGWFYDGKSEVRWSKTTTLQQTWGAMERLVDKGLARSIGVSNYQAQSVYDALIYARI
KPATLQIEHHPYLQQPDLVSLAQTEGIVVTAYSSFGPTGFMELDMPRAKSVAPLMDSPVIKAL
ADKHRRTPAQVLLRWATQRGIAVIPKTSRPEVMAQNLDNTSFDLDSEDLAKIADMDLNIRFNK
PTNYFSANKLYLFG

>sp|P37680|SGBE_ECOLI L-ribulose-5-phosphate 4-epimerase SgbE OS=Escherichia coli (strain K12) GN=sgbE PE=1 SV=1

MLEQLKADVLAANLALPAHHLVTFTWGNVSAVDETRQWMVIKPSGVEYDVMTADDMVVVEI
ASGKVVEGSKKPSSDTPTHLALYRRYAEIGGIVHTHSRHATIWSQAGLDLPAWGTTHADYFY
GAIPCTRQMTAEEINGEYEYQTGEVIIETFEERGRSPAQIPAVLVHSHGPFAWGKNAADAVH
NAVVLEECAYMGLFSRQLAPQLPAMQNELLDKHYLRKHGANAYYGQ

>sp|P39306|ULAF_ECOLI L-ribulose-5-phosphate 4-epimerase UlaF OS=Escherichia coli (strain K12) GN=ulaF PE=1 SV=1

MQKLKQQVFEANMELPRYGLVTFTWGNVSAIDRERGLVVIKPSGVAYETMKAADMVVVDMS
GKVVEGEYRPSSDTATHLELYRRYPSLGGIVHTHSTHATAWAQAGLAIPALGTTHADYFFGD
IPCTRGLSEEEVQGEYELNTGKVIIETLGNAEPLHTPGIVVYQHGPFAWGKDAHDAVHNAVV
MEEVAKMAWIARGINPQLNHIDSFLMNKHFMRKHGPNAYYGQK

>sp|P0AFP4|YBBO_ECOLI Uncharacterized oxidoreductase YbbO OS=Escherichia coli (strain K12) GN=ybbO PE=3 SV=1

MTHKATEILTGKVMQKSVLITGCSSGIGLESALELKRQGFHVLAGCRKPDDVERMNSMGFTG
VLIDLDSPESVDRAADEVIALTDNCLYGIFNNAGFGMYGPLSTISRAQMEQQFSANFFGAHQL
TMRLLPAMLPHGEGRIVMTSSVMGLISTPGRGAYAASKYALEAWSDALRMELRHSGIKVSLI
EPGPIRTRFTDNVNQTQSDKPVENPGIAARFTLGPEAVVDKVRHAFISEKPKMRYPVTLVTW
AVMVLKRLLPGRVMDKILQG

>tr|A2QBD7|A2QBD7_ASPNC Aspergillus niger contig An01c0480, genomic contig OS=Aspergillus niger (strain CBS 513.88 / FGSC A1513) GN=An01g14880 PE=4 SV=1

MSLPSHFTINTGAKIPAVGFGTWQAKPLEVENAVEVALREGYRHIDCAAIYRNETEVGNGIRK
SGVPREEIFITGKLWNTKHAPEDVEPALDKTLQDLGVAYLDLYLMHWPCAFKGGDKWFPLN
DDGVFDLANIDYITTYRAMEKLLATGKVRAIGVSNFNIRRLEELLGQVSVVPAVNQIEAHPYLQ
QPDLLQFCQSKGILIEAYSPLGNNQTGEPRTVDDPLVHRVAGELSLDPGPLLASWGVQRGT
VVLSKSVTPARIAANLRVRALPEGAFAQLNSLERHKRFNFPGHWGYDIFEEVGEEAVRQTAL
AAGPSNKVKFTV

>tr|Q9I1Q0|Q9I1Q0_PSEAE Probable aldehyde dehydrogenase OS=Pseudomonas aeruginosa (strain ATCC 15692 / DSM 22644 / CIP 104116 / JCM 14847 / LMG 12228 / 1C / PRS 101 / PAO1) GN=PA2217 PE=4 SV=1

MTAILGHNFIGGARSAAGTLFLQSLDAASGEALPYRFVQATPEEVDAAAEAAASAYPHYRQL
PASRRAEFLDTIAAELDALDDDFVAIVCRETALPATRIQGERSRTSGQLRLFAEVLRRGDFHG
ARIDRARPERKPLPRVDLRQCRIGLGPVAVFGASNFPLAFSTAGGDTAAALAAGCPVVFKAH
SGHMATAERVAAAILRAAERTGMPAGVFNMIYGGGVGERLVRHPAIQAVGFTGSLKGGRAL

CDMAAARAQPIPVFAEMSSINPVVLLPAALKKRGEAVADELSASVVLGCGQFCTNPGLVIGIR
SAQFSAFLERFAARMDDQPAQTMLNTGTLASYEKGLAALHAHPRVRHLAGQPQEGRQARP
QLFQADVSLLLEGDELLQEEVFGPASVVVEVADHAELKRALHGLHGQLTATLIAEAEDLASFA
DLVPLLEQKAGRLLLNGYPTGVEVCDAMVHGGPYPATSDARGTSVGTLAIDRFLRPVCYQN
YPDAWLPEALKDGNPLGIARLVDGIVTRAAVA

>tr|Q9I1Q1|Q9I1Q1_PSEAE Uncharacterized protein OS=Pseudomonas aeruginosa (strain ATCC 15692 / DSM 22644 / CIP 104116 / JCM 14847 / LMG 12228 / 1C / PRS 101 / PAO1) GN=PA2216 PE=4 SV=1

MRLIQFEDRAGQRRVGVVEGAGIQVLRGVRSTRELGLAAIRAGSGLHDEVLRRGSEPGPDY
AGLLEEGRVLPPLDHDDPAHCLVSGTGLTHLGSAATRDRMHQQNQGDETALTDTMRIFRW
GLEGGKPPAGQVGAQPEWFYKGDGGIVVRPGADFPLPAFAEDAGEEPELVGLYLIGDDRRP
YRLGYALGNEFSDHLMERRNYLYLAHSKLRACCYGPELRVGELPRHLQGESRILRDGEVLW
QQAFLSGEDNMCHSLENLEYHHFKYAQFLRPGDVHVHYFGTATLSFADGIKAAPGDVFEIAM
AEFGAPLRNGIAAVETALTPGRVVAL

>tr|Q97VG1|Q97VG1_SULSO Muconate cycloisomerase related protein OS=Sulfolobus solfataricus (strain ATCC 35092 / DSM 1617 / JCM 11322 / P2) GN=SSO2665 PE=4 SV=1

MTKISEIEAYILGKEVTSAQWASLMVLVRVTTNDGRVGWGETVSALRAEAVANFVKKINTVLK
GNDVFNVEKNRLEWYKHDFNMTISLESTTAYSAVDIASWDIIGKELGAPLYKLLGGKTRDKVL
VYANGWYQNCVKPEDFAEKAKEIVKMGYKALKFDPFGPYFNDISKKGLDIAEERVKAVREAV
GDNVDILIEHHGRFNANSAIMIAKRLEKYNPLFMEEPIHPEDVEGLRKYRNNTSLRIALGERIIN
KQQALYFMKEGLVDFLQADLYRIGGVTETKKVVGIAETFDVQMAFHNAQGPILNAVTLQFDA
FIPNFLIQESFYDWFPSWKRELIYNGTPIDNGYAIIPERPGLGVEVNEKMLDSLKVKGEEYFNP
EEPVWVVKGTWRDY

>sp|Q8X167|XKS1_ASPNG D-xylulose kinase A OS=Aspergillus niger GN=xkiA PE=1 SV=1

MQGPLYIGFDLSTQQLKGLVVNSDLKVVYVSKFDFDADSRGFPIKKGVLTNEAEHEVFAPVA
LWLQALDGVLEGLRKQGMDFSQIKGISGAGQQHGSVYWGENAEKLLKELDASKTLEEQLDG
AFSHPFSPNWQDSSTQKECDEFDAALGGQSELAFATGSKAHHRFTGPQIMRFQRKYPDVY
KKTSRISLVSSFIASLFLGHIAPMDISDVCGMNLWNIKKGAYDEKLLQLCAGSSGVDDLKRKL
GDVPEDGGIHLGPIDRYYVERYGFSPDCTIIPATGDNPATILALPLRASDAMVSLGTSTTFLMS
TPSYKPDPATHFFNHPTTAGLYMFMLCYKNGGLARELVRDAVNEKLGEKPSTSWANFDKVT
LETPPMGQKADSDPMKLGLFFPRPEIVPNLRSGQWRFDYNPKDGSLQPSNGGWDEPFDEA
RAIVESQMLSLRLRSRGLTQSPGEGIPAQPRRVYLVGGGSKNKAIAKVAGEILGGSEGVYKL
EIGDNACALGAAYKAVWAMERAEGQTFEDLIGKRWHEEEFIEKIADGYQPGVFERYGQAVE
GFEKMELEVLRQEGKH

>sp|A2Q8B5|XYL1_ASPNC Probable NAD(P)H-dependent D-xylose reductase xyl1 OS=Aspergillus niger (strain CBS 513.88 / FGSC A1513) GN=xyl1 PE=3 SV=1

MASPTVKLNSGYDMPLVGFGLWKVNNDTCADQIYHAIKEGYRLFDGACDYGNEVEAGQGIA
RAIKDGLVKREELFIVSKLWNSFHDGDRVEPICRKQLADWGIDYFDLYIVHFPISLKYVDPAVR
YPPGWKSEKDELEFGNATIQETWTAMESLVDKKLARSIGISNFSAQLVMDLLRYARIRPATLQ

IEHHPYLTQTRLVEYAQKEGLTVTAYSSFGPLSFLELSVQNAVDSPPLFEHQLVKSIAEKHGR
TPAQVLLRWATQRGIAVIPKSNNPQRLKQNLDVTGWNLEEEEIKAISGLDRGLRFNDPLGYG
LYAPIF

>sp|Q876L8|XYL1_HYPJE NAD(P)H-dependent D-xylose reductase xyl1 OS=Hypocrea jecorina GN=xyl1 PE=1 SV=1

MASPTLKLNSGYDMPQVGFGLWKVDNAVCADTVYNAIKAGYRLFDGACDYGNEKECGEGV
ARAIKDGLVKREDLFIVSKLWQTFHDEDKVEPITRRQLADWQIDYFDLFLVHFPAALEYVDPS
VRYPPGWFYDGKSEVRWSKTTTLQQTWGAMERLVDKGLARSIGVSNYQAQSVYDALIYARI
KPATLQIEHHPYLQQPDLVSLAQTEGIVVTAYSSFGPTGFMELDMPRAKSVAPLMDSPVIKAL
ADKHRRTPAQVLLRWATQRGIAVIPKTSRPEVMAQNLDNTSFDLDSEDLAKIADMDLNIRFNK
PTNYFSANKLYLFG

>sp|P09099|XYLB_ECOLI Xylulose kinase OS=Escherichia coli (strain K12) GN=xylB PE=1 SV=1

MYIGIDLGTSGVKVILLNEQGEVVAAQTEKLTVSRPHPLWSEQDPEQWWQATDRAMKALGD
QHSLQDVKALGIAGQMHGATLLDAQQRVLRPAILWNDGRCAQECTLLEARVPQSRVITGNL
MMPGFTAPKLLWVQRHEPEIFRQIDKVLLPKDYLRLRMTGEFASDMSDAAGTMWLDVAKRD
WSDVMLQACDLSRDQMPALYEGSEITGALLPEVAKAWGMATVPVVAGGGDNAAGAVGVG
MVDANQAMLSLGTSGVYFAVSEGFLSKPESAVHSFCHALPQRWHLMSVMLSAASCLDWAA
KLTGLSNVPALIAAAQQADESAEPVWFLPYLSGERTPHNNPQAKGVFFGLTHQHGPNELAR
AVLEGVGYALADGMDVVHACGIKPQSVTLIGGGARSEYWRQMLADISGQQLDYRTGGDVG
PALGAARLAQIAANPEKSLIELLPQLPLEQSHLPDAQRYAAYQPRRETFRRLYQQLLPLMA

>sp|P00944|XYLA_ECOLI Xylose isomerase OS=Escherichia coli (strain K12) GN=xylA PE=3 SV=1

MQAYFDQLDRVRYEGSKSSNPLAFRHYNPDELVLGKRMEEHLRFAACYWHTFCWNGADM
FGVGAFNRPWQQPGEALALAKRKADVAFEFFHKLHVPFYCFHDVDVSPEGASLKEYINNFA
QMVDVLAGKQEESGVKLLWGTANCFTNPRYGAGAATNPDPEVFSWAATQVVTAMEATHKL
GGENYVLWGGREGYETLLNTDLRQEREQLGRFMQMVVEHKHKIGFQGTLLIEPKPQEPTKH
QYDYDAATVYGFLKQFGLEKEIKLNIEANHATLAGHSFHHEIATAIALGLFGSVDANRGDAQL
GWDTDQFPNSVEENALVMYEILKAGGFTTGGLNFDAKVRRQSTDKYDLFYGHIGAMDTMAL
ALKIAARMIEDGELDKRIAQRYSGWNSELGQQILKGQMSLADLAKYAQEHHLSPVHQSGRQ
EQLENLVNHYLFDK

>sp|Q3SYZ6|XYLB_BOVIN Xylulose kinase OS=Bos taurus GN=XYLB PE=2 SV=1

MAERAARHCCLGWDFSTQQVKVVAVDAELSVFYEDSVHFDRDLVEFGTQGGVHVHKDGLT
VTSPVLMWVQALDIILEKMKASGFDFSQVLALSGAGQQHGSVYWKTGASQVLTSLSPDLPL
REQLQACFSISNCPVWMDSSTAAQCRQLEAAVGGAQALSLLTGSRAYERFTGNQIAKIYQQ
NPEAYSHTERISLVSSFAASLFLGSYSPVDYSDGSGMNLLQIQDKVWSQACLGACAPRLEEK
LGRPVPSCSIVGAISSYFVQRYGFPPECKVVAFTGDNPASLAGMRLEEGDIAVSLGTSDTLFL
WLQEPTPALEGHIFCNPVDPQHYMALLCFKNGSLMREKIRDESASGSWSKFSKALQSTGMG

NSGNLGFYFDVMEITPEIIGRHRFTAENHEVSAFPQDVEIRALIEGQFMAKKIHAEALGYRVM
PKTKILATGGASHNRDILQVLADVFGAPVYVIDTANSACVGSAYRAFHGPSLLCLVSIY

>sp|P0AFP4|YBBO_ECOLI Uncharacterized oxidoreductase YbbO OS=Escherichia coli (strain K12) GN=ybbO PE=3 SV=1

MTHKATEILTGKVMQKSVLITGCSSGIGLESALELKRQGFHVLAGCRKPDDVERMNSMGFTG
VLIDLDSPESVDRAADEVIALTDNCLYGIFNNAGFGMYGPLSTISRAQMEQQFSANFFGAHQL
TMRLLPAMLPHGEGRIVMTSSVMGLISTPGRGAYAASKYALEAWSDALRMELRHSGIKVSLI
EPGPIRTRFTDNVNQTQSDKPVENPGIAARFTLGPEAVVDKVRHAFISEKPKMRYPVTLVTW
AVMVLKRLLPGRVMDKILQG

>Ss-LADH_tr|A3LNE3|A3LNE3_PICST Aldehyde dehydrogenase OS=Scheffersomyces stipitis (strain ATCC 58785 / CBS 6054 / NBRC 10063 / NRRL Y-11545) OX=322104 GN=ALD5 PE=3 SV=1

MSLPLFVPIKLPNGTTYEQPTGLFINNEFVQSKSKKTFGTVSPSTEEEITQVYEAFSEDIDDAV
EAATAAFHSSWSTSDPQVRMKVLYKLADLIDEHADTLAHIEALDNGKSLMCSKGDVALTAAY
FRSCAGWTDKIKGSVIETGDTHFNYTRREPIGVCGQIIPWNFPLLMASWKLGPVLCTGCTTVL
KTAESTPLSALYLASLIKEAGAPPGVVNVVSGFGPTAGAPISSHPKIKKVAFTGSTATGRHIMK
AAAESNLKKVTLELGGKSPNIVFDDADVKSTIQHLVTGIFYNTGEVCCAGSRIYVQEGIYDKIV
SEFKNAAESLKIGDPFKEDTFMGAQTSQLQLDKILKYIDIGKKEGATVITGGERFGNKGYFIKP
TIFGDVKEDHQIVRDEIFGPVVTITKFKTVEEVIALANDSEYGLAAGVHTTNLSTAISVSNKINS
GTIWVNTYNDFHPMVPFGGYSQSGIGREMGEEALDNYTQVKAVRIGLSQ

>Ss-LRA1_sp|A3LZU7|RM1DH_PICST L-rhamnose-1-dehydrogenase OS=Scheffersomyces stipitis (strain ATCC 58785 / CBS 6054 / NBRC 10063 / NRRL Y-11545) OX=322104 GN=DHG2 PE=1 SV=2

MTGLLNGKVVAITGGVTGIGRAIAIEMARNGAKVVVNHLPSEEQAQLAKELKEEISDGENNVL
TIPGDISLPETGRRIVELAVEKFGEINVFVSNAGVCGFREFLEITPETLFQTVNINLNGAFFAIQA
AAQQMVKQGKGGSIIGISSISALVGGAHQTHYTPTKAGILSLMQSTACALGKYGIRCNAILPGT
ISTALNEEDLKDPEKRKYMEGRIPLGRVGDPKDIAGPAIFLASDMSNYVNGAQLLVDGGLFVN
LQ

>Dh-LRA2_tr|Q6BQZ8|Q6BQZ8_DEBHA DEHA2E01078p OS=Debaryomyces hansenii (strain ATCC 36239 / CBS 767 / JCM 1990 / NBRC 0083 / IGC 2968) OX=284592 GN=DEHA2E01078g PE=4 SV=2

MPSKKYKIIDSHVHLFAKRNFKLLKFDEAHPLHSDFRLDEYLKYSMNEEFQIDGLVFIETDPIA
DLSKALEGCEYPIQEYLYVARNITGNLLPDEGETSELKQNFIKAIVPWAPMPLGKSSVSSYVE
MLKSRSSTDEFNLVKGFRYLVQDKLPNTMLQRDFVESLKWLDDNNFIFDWGIDLRCGGLWQ
FEETIEVLKQVPNLKYVINHLTKPNLSIDPTKIEENDEFLQWKNYMKQIFVNSPNSYMKLSGGF
SELPSEIIENRDKCAEYIYPWFKVCFDLWNVDRTIWASNWPVCTLTAGEDLTSKWFEVTEML
FDKIELNEESRKKIYGTNYLKAYNLI

>Ss-LRA4_tr|A3LZU9|A3LZU9_PICST L-KDR aldolase OS=Scheffersomyces stipitis (strain ATCC 58785 / CBS 6054 / NBRC 10063 / NRRL Y-11545) OX=322104 GN=PICST_64442 PE=3 SV=1

MTISAALPKRGVYTPVPTFFKKDLHTIDYDSQIEHAKFLQQNGITGLVLLGSTGENSHLTRKER
IELVSTIHEELPDFPLMAGVAQNSVEDAIEEILQLKNAGAQHALVLPSSYFGASIKQQGIIDWYT
EVADNASLPVLIYVYPGVSNNISIDPRTIKKLSAHPNIVGAKISHGDVSHHAIIGLDQEIAANQFI
TLTGLGQILLPVLVVGIQGTVDALCGAFPKIYVKLLENYDKGDLRAAAELQLVISRAEELVVKF
GVVGIKKAIHFATGIGETYLGRAPLTQDVNDADWKSYNDYLLGIVSVESTL

>sp|Q38707|MTDH_APIGR Mannitol dehydrogenase OS=Apium graveolens OX=4045 GN=MTD PE=1 SV=1

MAKSSEIEHPVKAFGWAARDTTGLLSPFKFSRRATGEKDVRLKVLFCGVCHSDHHMIHNNW
GFTTYPIVPGHEIVGVVTEVGSKVEKVKVGDNVGIGCLVGSCRSCESCCDNRESHCENTIDT
YGSIYFDGTMTHGGYSDTMVADEHFILRWPKNLPLDSGAPLLCAGITTYSPLKYYGLDKPGT
KIGVVGLGGLGHVAVKMAKAFGAQVTVIDISESKRKEALEKLGADSFLLNSDQEQMKGARSS
LDGIIDTVPVNHPLAPLFDLLKPNGKLVMVGAPEKPFELPVFSLLKGRKLLGGTINGGIKETQE
MLDFAAKHNITADVEVIPMDYVNTAMERLVKSDVRYRFVIDIANTMRTEESLGA

>Ss_agaK_sp|A0KYQ6|AGAK_SHESA N-acetylgalactosamine kinase AgaK OS=Shewanella sp. (strain ANA-3) OX=94122 GN=agaK PE=1 SV=1

MYYGLDIGGTKIELAIFDTQLALQDKWRLSTPGQDYSAFMATLAEQIEKADQQCGERGTVGIA
LPGVVKADGTVISSNVPCLNQRRVAHDLAQLLNRTVAIGNDCRCFALSEAVLGVGRGYSRVL
GMILGTGTGGGLCIDGKLYLGANRLAGEFGHQGVSANVACRHQLPLYVCGCGLEGCAETYV
SGTGLGRLYQDIAGQTADTFAWLNALRCNDPLAIKTFDTYMDILGSLMASLVLAMDPDIIVLG
GGLSEVEEILAALPQATKAHLFDGVTLPQFKLADFGSASGVRGAALLGHGLDAGISYEA

>Ss_agaA_sp|A0KYQ5|AGAA2_SHESA N-acetylgalactosamine-6-phosphate deacetylase OS=Shewanella sp. (strain ANA-3) OX=94122 GN=agaAII PE=1 SV=1

MKPNTDFMLIADGAKVLTQGNLTEHCAIEVSDGIICGLKSTISAEWTADKPHYRLTSGTLVAG
FIDTQVNGGGGLMFNHVPTLETLRLMMQAHRQFGTTAMLPTVITDDIEVMQAAADAVAEAID
CQVPGIIGIHFEGPHLSVAKRGCHPPAHLRGITEREWLLYLRQDLGVRLITLAPESVTPEQIKR
LVASGAIISLGHSNADGETVLKAIEAGASGFTHLYNGMSALTSREPGMVGAAFASENTYCGIIL
DGQHVHPISALAAWRAKGTEHLMLVTDAMSPLGSDQTEFQFFDGKVVREGMTLRDQHGSL
AGSVLDMASAVRYAATELNLGLSNAVQMATRTPAEFIQRPQLGDIAEGKQADWVWLDDDQ
RVLAVWIAGELLYQAEQARFA

>Ss_agaS_sp|A0KYQ7|AGAS_SHESA D-galactosamine-6-phosphate deaminase AgaS OS=Shewanella sp. (strain ANA-3) OX=94122 GN=agaS PE=1 SV=1

MLTSPLSPFEHEDSNLLLSAEQLTQYGAFWTAKEISQQPKMWRKVSEQHSDNRTIAAWLTPI
LAKPQLRIILTGAGTSAYIGDVLAAHIQQHLPLATQQVEAISTTDIVSHPELYLRGNIPTLLISYG
RSGNSPESMAAVELAEQLVDDCYHLAITCNGQGKLANYCADKSHCYLYKLPDETHDVSFAM
TSSFTCMYLATLLIFAPNSQALMQCIEMAEHILTERLADIRLQSEQPSKRVVFLGGGPLKAIAQ

EAALKYLELTAGQVVSAFESPLGFRHGPKSLVDSHTQVLVMMSSDPYTRQYDNDLIQELKRD
NQALSVLTLSEELLTGSSGLNEVWLGLPFILWCQILAIYKAIQLKVSPDNPCPTGQVNRVVQG
VNVYPFVK

>AAA93234.2 amygdalin hydrolase isoform AH I precursor [Prunus serotina]

MATKLGSLLLCALLLAGFALTNSKAAKTDPPIHCASLNRSSFDALEPGFIFGTASAAYQFEGA
AKEDGRGPSIWDTYTHNHSERIKDGSNGDVAVDQYHRYKEDVRIMKKMGFDAYRFSISWSR
VLPNGKISGGVNEDGIKFYNNLINEILRNGLKPFVTIYHWDLPQALEDEYGGFLSPNIVDHFRD
YANLCFKKFGDRVKHWITLNEPYTFSSSGYAYGVHAPGRCSAWQKLNCTGGNSATEPYLVT
HHQLLAHAAAVKLYKDEYQASQNGLIGITLVSPWFEPASEAEEDINAAFRSLDFIFGWFMDPL
TNGNYPHLMRSIVGERLPNFTEEQSKLLKGSFDFIGLNYYTTRYASNAPKITSVHASYITDPQ
VNATAELKGVPIGPMAASGWLYVYPKGIHDLVLYTKEKYNDPLIYITENGVDEFNDPKLSMEE
ALKDTNRIDFYYRHLCYLQAAIKKGSKVKGYFAWSFLDNFEWDAGYTVRFGINYVDYNDNLK
RHSKLSTYWFTSFLKKYERSTKEIQMFVESKLEHQKFESQMMNKVQSSLAVVV

>ACO22019.1 beta-glucosidase (Gentiobiase) (Cellobiase) (Beta-D-glucoside glucohydrolase) (Amygdalase) [Streptococcus pneumoniae P1031]

MTIFPDDFLWGGAVAANQVEGAYNEDGKGLSVQDVLPKGGLGEATENPTEDNLKLIGIDFYH
KYKEDISLFSEMGFNVFRTSIAWSRIFPKGDEEEPNEAGLKYYDELFDELHAHGIEPLVTLSH
YETPLYLARKYHGWIDRRMIHFYEKFARTVLERYKDKVKYWLTFNEVNSVLELPFTSGGIDIP
KENLSKQELYQAIHHELVASSLVTKIAREINSEFKVGCMVLAMPAYPMTPNPKDVWATHEYE
NLNYLFSDVHVRGYYPNYAKRYFKENDINIEFAAEDAELLKNYTVDFLSFSYYMSVTQSALPT
QYNSGEGNIIGGLVNPYLESSEWGWQIDPIGLRIILNRYYDRYQIPLFIVENGLGAKDQLIKDEF
NNLTVQDDYRIQYMKEHLLQVAEALQDGVEIMGYTSWGCIDCVSMSTAQLSKRYGLIYVDRN
DDGNGTFNRYKKMSFTWYKGVIESNGESLFK

>ACK40981.1 beta-glucosidase (Gentiobiase) (Cellobiase) (Beta-D-glucoside glucohydrolase) (Amygdalase) [Listeria monocytogenes HCC23]

MTESKFPKGFLWGGAVAANQCEGAYLEDGKGLSLVDILPTVEDGRWEALFNPSKALATDYG
FYPSHESIDFYHRYKEDIKLMAEMGFKCFRMSISWPRIFPNGDETTPNEKGLAFYDAVFDEC
HKYGIEPVVTINHFDTPLEVFKKYGGWKNRKCIDFYLNFCEAIFTRYKDKVKYWMTFNEINMIL
HLPYIGGGLDVTKEANPEEVKYQAAHHQLVASALATKLGHEINPENQIGCMLAAGNTYPMTC
NPKDVWKSIEADREGYFFIDVQARGYYPSYTKRFFKEHNINIKMEDGDLDALRDHTVDYVAF
SYYSSRLTSADPEKNKETEGNVFATLKNPYLKASEWGWQIDPLGLRITMNTIYDRYQKPLFIV
ENGLGAVDTVAEDGSITDDYRIDYMREHVREMGEAIEDGVELLGYTPWGCIDLVSAGSGEM
KKRYGFIYVDRDNKGNGTLNRSKKKSFDWYKKVIETNGKDID

>D-ribose_pyranase sp|P04982|RBSD_ECOLI D-ribose pyranase OS=Escherichia coli (strain K12) OX=83333 GN=rbsD PE=1 SV=3

MKKGTVLNSDISSVISRLGHTDTLVVCDAGLPIPKSTTRIDMALTQGVPSFMQVLGVVTNEMQ
VEAAIIAEEIKHHNPQLHETLLTHLEQLQKHQGNTIEIRYTTHEQFKQQTAESQAVIRSGECSP
YANIILCAGVTF

>ribokinase_S_cerevisiae EDN62156.1 ribokinase [Saccharomyces cerevisiae YJM789]

MGITVIGSLNYDLDTFTDRLPNAGETFRANHFETHAGGKGLNQAAAIGKLKNPSSRYSVRMI
GNVGNDTFGKQLKDTLSDCGVDITHVGTYEGINTGTATILIEEKAGGQNRILIVEGANSKTIYD
SKQLCEIFPEGKEEEEYVVFQHEIPDPLSIIKWIHANRPNFQIVYNPSPFKAMRKKDWELVDLL
VVNEIEGLQIVESVFDNELVEEIREKIKDDFLGEYRKICELLYEKLMNRKKRGIVVMTLGSKGV
LFCSHESPEVQFLPAIENVSVVDTTGAGDTFLGGLVTQLYQGETLSTAIKFSTLASSLTIQRKG
AAESMPLYKDVQKDA

>phosphoribomutase_S_cerevisiae NP_014005.1 phosphoribomutase PRM15
[Saccharomyces cerevisiae S288C]

MLQGILETVPSDLKDPISLWFKQDRNPKTIEEVTALCKKSDWNELHKRFDSRIQFGTAGLRSQ
MQAGFSRMNTLVVIQASQGLATYVRQQFPDNLVAVVGHDHRFHSKEFARATAAAFLLKGFK
VHYLNPDHEFVHTPLVPFAVDKLKASVGVMITASHNPKMDNGYKVYYSNGCQIIPPHDHAIS
DSIDANLEPWANVWDFDDVLNKALKQGKLMYSREEMLKLYLEEVSKNLVEINPLKLEVKAKP
WFVYTPMHGVGFDIFSTIVKKTLCLVEGKDYLCVPEQQNPDPSFPTVGFPNPEEKGALDIGIN
LAEKHDIDLLVANDPDADRFSVAVKDMQSGEWRQLTGNEIGFLFAFYEYQKYKSMDKEFQH
VHPLAMLNSTVSSQMIKKMAEIEGFHYEDTLTGFKWIGNRAILLEKKGYYVPFGFEEAIGYMF
PAMEHDKDGISASIVFLQAYCKWKIDHNLDPLNVLENGFKKYGVFKEYNGYYVVPNPTVTKDI
FDYIRNVYTPEGASYPSSIGEEIEVLYYRDLTTGYQSDTINHKPTLPVDPTSQMITVSARPSNG
SENEHIRFTIRGSGTEPKLKVYIEACANEEQRASFLAKLTWNVLRREWFRPDEMNIVTKF

>Acetaldehyde_dehydrogenase_S_cerevisiae AAB68304.1 Ald6p: Acetaldehyde
dehydrogenase [Saccharomyces cerevisiae]

MTKLHFDTAEPVKITLPNGLTYEQPTGLFINNKFMKAQDGKTYPVEDPSTENTVCEVSSATTE
DVEYAIECADRAFHDTEWATQDPRERGRLLSKLADELESQIDLVSSIEALDNGKTLALARGDV
TIAINCLRDAAAYADKVNGRTINTGDGYMNFTTLEPIGVCGQIIPWNFPIMMLAWKIAPALAMG
NVCILKPAAVTPLNALYFASLCKKVGIPAGVVNIVPGPGRTVGAALTNDPRIRKLAFTGSTEVG
KSVAVDSSESNLKKITLELGGKSAHLVFDDANIKKTLPNLVNGIFKNAGQICSSGSRIYVQEGI
YDELLAAFKAYLETEIKVGNPFDKANFQGAITNRQQFDTIMNYIDIGKKEGAKILTGGEKVGDK
GYFIRPTVFYDVNEDMRIVKEEIFGPVVTVAKFKTLEEGVEMANSSEFGLGSGIETESLSTGL
KVAKMLKAGTVWINTYNDFDSRVPFGGVKQSGYGREMGEEVYHAYTEVKAVRIKL

>deoxyribose-phosphate_aldolase_P_lactucaedebilis ORY78125.1 deoxyribose-phosphate
aldolase [Protomyces lactucaedebilis]

MNRLIDHTILKPDATKAEVEKICDEALALETATVCVNTRWLPLVSKKLANSNVLPIAVVGFPLG
ACLTEAKVFETKLAIQQGAKEIDMVIDVGALKDGEVEHVEKDIHAVVQAAGNIPVKVILETCLLT
DEQKRTACKLCKKAGAAFVKTSTGFSKSGATVADTKLMREEVGKEMGVKASGGIRTFKDAQ
AMVDAGASRIGASASVAIMAEANASSNV

>aldehyde-alcohol_dehydrogenase_Fusarium KLP10254.1 aldehyde-alcohol dehydrogenase
[Fusarium fujikuroi]

MTDTLKPYRMSQLEGGHRPGSDAIFNSHSKDGILSALKEWNSQRILLVHSKALAKNTHVISYL
KEALGDRLTNVKEGVGSHSPYSDVIDIAHRITEHKIDCVISVGSGSYSDACKVARLMSATLPA
GFREEDMENLLDQDKGVTPQDKMKKADGVKLILVPTSLSAGEWNHTASCTNSAGKKQHFSL
QDGGAPDLILMDPWVARTSPEKLWMSSGIRAVDHCVETLCNPECKKYPDVQEWCEEALRD
LAKGLVEYKEGLGRGKEGEDELVHGVSKCQTGSRMALMGFIIYRVNMGASHAIGHQLGSVG
KVMHGITSCIMLPPVLRYTKARNPGAQARIVEIFNEALGWEETDASDCVARLVEVTGLPSTLR
DVGVTNNEQIEQIIDKTMTDVMFSFGKILTRKEVSEIVYSTK
